# A Randomized Phase 1 Clinical Trial of a Respiratory Syncytial Virus and Human Metapneumovirus Combination Protein-Based Virus-like Particle Vaccine in Adults 60–75 Years of Age

**DOI:** 10.1093/ofid/ofaf160

**Published:** 2025-03-13

**Authors:** Craig Shapiro, Nelia Sánchez-Crespo, Max Ciarlet, Nicholas Hourguettes, Judy Wen, Wasima Rida, Jennifer Price, April E Engram, Elizabeth M Adams, Niranjan Kanesa-thasan

**Affiliations:** Cenexel RCA, Hollywood, Florida, USA; Cenexel RCA, Hollywood, Florida, USA; Icosavax, Vaccines and Immune Therapies, BioPharmaceuticals R&D, AstraZeneca, Seattle, Washington, USA; Icosavax, Vaccines and Immune Therapies, BioPharmaceuticals R&D, AstraZeneca, Seattle, Washington, USA; Icosavax, Vaccines and Immune Therapies, BioPharmaceuticals R&D, AstraZeneca, Seattle, Washington, USA; Icosavax, Vaccines and Immune Therapies, BioPharmaceuticals R&D, AstraZeneca, Seattle, Washington, USA; Icosavax, Vaccines and Immune Therapies, BioPharmaceuticals R&D, AstraZeneca, Seattle, Washington, USA; Icosavax, Vaccines and Immune Therapies, BioPharmaceuticals R&D, AstraZeneca, Seattle, Washington, USA; Icosavax, Vaccines and Immune Therapies, BioPharmaceuticals R&D, AstraZeneca, Seattle, Washington, USA; Icosavax, Vaccines and Immune Therapies, BioPharmaceuticals R&D, AstraZeneca, Seattle, Washington, USA

**Keywords:** human metapneumovirus, IVX-A12, respiratory syncytial virus, vaccines, virus-like particle

## Abstract

**Background:**

Respiratory syncytial virus (RSV) and human metapneumovirus (hMPV) are important causes of severe lower respiratory tract disease (LRTD) in adults ≥65 years of age. We report safety and immunogenicity for IVX-A12, a combination, protein-based virus-like particle (VLP) vaccine against RSV- and hMPV-associated LRTD.

**Methods:**

This phase 1 trial (NCT05664334) randomized healthy adults 60–75 years of age to receive 1 intramuscular dose of IVX-A12 at low (75 µg RSV/75 µg hMPV), medium (75 µg RSV/150 µg hMPV), or high dose levels (75 µg RSV/225 µg hMPV) ± oil-in-water adjuvant MF59® (low and medium dose levels only), or placebo. Safety and immunogenicity were assessed through day 365 postvaccination.

**Results:**

Overall, 140 participants received IVX-A12 (n = 117) or placebo (n = 23). Solicited adverse reactions (ARs) were transient, with mild to moderate severity; ARs were reported by 47.0% (n = 55/117) and 34.8% (n = 8/23) of IVX-A12 and placebo recipients, respectively. There were no vaccine-related serious adverse events. All IVX-A12 dose levels and formulations boosted preexisting RSV- and hMPV-specific neutralizing antibody (nAb) responses; from baseline to day 28, nAb titers against RSV A or B increased up to 4- or 3-fold, and nAb titers against hMPV A or B increased up to 5- or 4-fold, respectively. Neutralizing responses in IVX-A12 recipients through day 365 were maintained above or around baseline and exceeded those in placebo recipients.

**Conclusions:**

IVX-A12 was well-tolerated and elicited RSV- and hMPV-specific antibody responses in adults 60–75 years of age. These data support the ongoing clinical development of the RSV/hMPV combination VLP vaccine IVX-A12.

## Teaser

IVX-A12 is an investigational, combination, protein-based virus-like particle vaccine against respiratory syncytial virus (RSV) and human metapneumovirus (hMPV). IVX-A12 was well-tolerated and elicited RSV- and hMPV-specific antibody responses in adults 60–75 years of age, supporting further clinical evaluation.

Respiratory syncytial virus (RSV) is a major cause of lower respiratory tract disease (LRTD) worldwide, with seasonal epidemics contributing to substantial morbidity and mortality, particularly in older adults [1], immunocompromised individuals [2], and those with chronic comorbidities [3]. The impact of RSV is well characterized [2–5], accounting for an estimated >175 000 hospitalizations and 14 000 deaths each year among adults ≥65 years of age in the United States (US) alone [2, 3]. RSV is a single-strand, negative-sense RNA virus that belongs to the family Pneumoviridae and has 2 distinct subgroups: RSV A and RSV B [6]. The most closely related human pathogen to RSV is human metapneumovirus (hMPV), which was first identified in the Netherlands in 2001 [7] and also has 2 subgroups: hMPV A and hMPV B [6]. While the burden of hMPV is less well known, hMPV is responsible for a significant proportion of LRTD in older adults; a recent study in adults hospitalized with respiratory viruses showed that hMPV was responsible for 8% of infections and RSV for 10%, with incidence increasing with age [8].

Infection with RSV or hMPV induces neutralizing antibodies (nAbs) that primarily target the prefusion conformation of the viral fusion protein (pre-F) [9] and that are known to protect against near-term reinfection in animal models and human studies [10, 11]. Structure-based design methods were therefore used to stabilize both RSV and hMPV pre-F for evaluation as vaccine antigens [12, 13]. At least 3 RSV vaccines have demonstrated efficacy in preventing RSV-associated LRTD and are now licensed for use in adults ≥60 years of age; RSVpreF3 and RSVpreF vaccines both contain recombinant RSV pre-F, and mRNA-1345 contains nucleoside-modified messenger RNA (mRNA), which encodes RSV pre-F in vivo [14–16]. However, there is no licensed hMPV vaccine currently available. The success of vaccines based on RSV pre-F suggests that vaccination with hMPV pre-F may also provide protection against hMPV-associated LRTD.

IVX-A12 is an investigational, computationally designed, combination vaccine containing 2 virus-like particles (VLPs) to induce immune responses against RSV and hMPV and prevent associated LRTD [17]. VLPs are nanoscale structures with a high-density, mono- or multimeric display of viral antigens, closely resembling the structure of a virus; VLPs do not contain genetic material and are not capable of infecting host cells [18, 19]. The 2 VLPs within IVX-A12 are comprised of a common, protein-based structural core, and each displays 20 copies of either RSV or hMPV pre-F trimers [17]. A previous phase 1 trial investigated varying dose levels of the RSV component of IVX-A12 and the 75 μg RSV VLP dose level was chosen for the next study [20]. This phase 1 trial evaluated the first-in-human safety and immunogenicity of IVX-A12 at the fixed 75 μg RSV VLP dose level and varying hMPV VLP dose levels, with and without adjuvant, in adults 60–75 years of age in the US.

## METHODS

### Trial Design

This phase 1, randomized, observer-blind, placebo-controlled, multicenter trial (NCT05664334) assessed the safety and immunogenicity of IVX-A12 at 4 sites across the US between September 2022 and January 2024.

Three dose levels of IVX-A12 containing a fixed amount of the RSV VLP (75 µg RSV VLP) and increasing amounts of the hMPV VLP were evaluated using a stepwise dose-escalation approach across 3 cohorts (Supplementary Figure 1). The low (75 µg RSV/75 µg hMPV) and medium (75 µg RSV/150 µg hMPV) dose levels were tested with and without MF59® (oil-in-water adjuvant; CSL Seqirus, Melbourne, Australia); the high dose level (75 µg RSV/225 µg hMPV) was tested without adjuvant. In cohort 1, participants were enrolled and randomized 5:1 to receive either unadjuvanted low dose level IVX-A12 or placebo. In cohorts 2 and 3, participants were enrolled and randomized 5:5:2 between 3 groups: adjuvanted low dose level IVX-A12, unadjuvanted medium dose level IVX-A12, and placebo in cohort 2; and adjuvanted medium dose level IVX-A12, unadjuvanted high dose level IVX-A12, and placebo in cohort 3. Initially, sentinel participants in each cohort were vaccinated, and if no stopping rules (Supplementary Methods) were triggered within 36 hours, the rest of the cohort was vaccinated.

The protocol and all amendments were approved by the institutional review board Advarra. The trial was performed in accordance with the principles of the Declaration of Helsinki and the International Council for Harmonisation guidelines for Good Clinical Practice, applicable regulatory requirements, and AstraZeneca's policy on bioethics.

### Participants

Healthy adults 60–75 years of age, including those with stable, well-controlled, chronic conditions without clinical exacerbation within the previous 12 months, were enrolled. Key exclusion criteria were prior receipt of any investigational RSV or hMPV vaccine; self-reported, laboratory-confirmed severe RSV or hMPV infection within the previous 12 months; high-risk comorbidities for RSV and hMPV; acute or chronic progressive unstable or uncontrolled medical conditions; frailty; and immunosuppression. A full list of eligibility criteria is provided in the Supplementary Methods. All participants provided written informed consent.

### Randomization and Masking

An interactive web randomization system ensured appropriate distribution of participants among groups. Randomization assignment was known only to the trial personnel who prepared and administered the vaccines at trial sites. All participants, data collectors (eg, the investigator), and data evaluators were masked.

### Vaccine

A single 0.5 mL dose of IVX-A12 with or without adjuvant, or placebo, was administered to participants on day 0 via deltoid intramuscular injection. Unadjuvanted IVX-A12 was formulated with sterile aqueous diluent, while adjuvanted IVX-A12 was formulated with MF59® emulsion (containing 9.75 mg squalene). Diluent was also used as placebo.

### Safety Assessments

Predefined injection site and systemic reactions were collected as solicited adverse reactions (ARs) for 7 days after vaccination (days 0–6). Unsolicited adverse events (AEs) were collected through day 28, and serious AEs (SAEs), medically attended AEs (MAAEs), adverse events of special interest (AESIs), and AEs leading to trial discontinuation were collected to the end of the trial (day 365). The only prespecified AESI was a clinical event of special interest of moderate to severe (grade 2–3) lower respiratory tract infection (LRTI) occurring postvaccination, as defined in the Supplementary Methods.

Solicited ARs were recorded and graded for severity via electronic diaries, which prompted participants to complete questions. Unsolicited AEs were reported at scheduled visits on days 0, 7, and 28, and via telephone on days 3 and 14, then were assessed for causality (related or not related to trial investigational product) and severity by the investigator. Details of severity gradings are provided in the Supplementary Methods. SAEs, MAAEs, AESIs, and AEs leading to trial discontinuation were assessed at all scheduled visits (days 0, 7, 28, 180, and 365) and telephone calls (days 3, 14, 56, 135, and 285). In addition, safety data were reviewed by an independent safety monitoring committee and the trial only continued or escalated to the next cohort if no stopping rules (Supplementary Methods) were triggered.

### Immunogenicity Assessments

Serum samples for immunogenicity testing were collected at scheduled visits on days 0 (prior to vaccination), 7, 28, 180, and 365. Serological assessments included virus neutralization assays using RSV A2, RSV B 18537, hMPV A1 NL/1/00, and hMPV B1 NL/1/99 to determine RSV A/B– and hMPV A/B–specific nAb titers. Enzyme-linked immunosorbent assays were also performed to evaluate RSV- and hMPV-specific pre-F immunoglobulin G (IgG), as well as VLP core-specific IgG. Serological assessments were carried out as described in the Supplementary Methods.

### Outcomes

Primary safety outcomes were incidence of injection site and systemic ARs through 7 days after vaccination, and incidence of unsolicited AEs through 28 days after vaccination. Secondary safety outcomes assessed SAEs, MAAEs, AESIs, and AEs leading to trial discontinuation through trial end (day 365).

Primary immunogenicity outcomes evaluated RSV- and hMPV-specific nAbs and pre-F IgG at day 28, through geometric mean titers (GMTs) and geometric mean fold rises (GMFRs) from baseline. The percentage of participants with a ≥4-fold increase in nAb titers from baseline (defined as the seroresponse rate [SRR]), as well as those with an ≥8-fold increase, was determined at day 28. Secondary immunogenicity outcomes included GMTs and GMFRs of RSV- and hMPV-specific nAbs and pre-F IgG at each additional visit through trial end (days 7, 180, and 365). SRRs and the percentage of participants with a ≥8-fold increase in nAb titers from baseline were also determined for each additional visit. Geometric mean ratios (GMRs) of the fold rise in pre-F IgG titers from baseline over fold rise in nAb titers from baseline were generated for each timepoint. An exploratory analysis assessed GMTs and GMFRs of VLP core-specific IgG at each timepoint (not including day 7) through day 365.

### Statistical Analysis

Enrollment of 20 participants per vaccine group provided a ≥90% probability of correctly selecting the most immunogenic IVX-A12 dose level and formulation, based on the mean day 28 log_2_-transformed hMPV A nAb response when the true standardized difference between the most and next most immunogenic group was at least 0.6 [21]. For example, if the standard deviation is 1.67 log_2_, a standardized difference of 0.6 implies a 2-fold increase in titers between the groups.

Safety analyses were performed on the safety set, defined as all participants who received IVX-A12 or placebo. Safety data were summarized descriptively by dose level/adjuvant formulation received.

Immunogenicity analyses were performed on the per-protocol set, defined as all participants who received IVX-A12 or placebo and had no major protocol deviations that could impact immunogenicity results for day 28. Participants were analyzed as randomized and were excluded if outcome data were missing; no missing data were imputed. Data for participants who had major protocol deviations affecting immunogenicity outcomes or RSV/hMPV infections after day 28 were excluded for subsequent timepoints. Immunogenicity data were primarily summarized descriptively with GMTs and GMFRs calculated by vaccine group (unadjuvanted low dose; low dose + MF59®; unadjuvanted medium dose; medium dose + MF59®; unadjuvanted high dose; and placebo), by pooled dose level (low dose ± MF59®; medium dose ± MF59®; unadjuvanted high dose; and placebo), and by baseline nAb titer tertile. Adjusted GMTs and GMFRs were also calculated after adjusting for baseline titers and sex. The GMR of the fold rise in pre-F IgG to the fold rise in nAb at day 28 was calculated by vaccine group for RSV A/B and for hMPV A/B.

Linear regression analyses were performed to assess the effects of adjuvant and dose level on RSV- and hMPV-specific nAb and pre-F IgG titers at day 28 after adjusting for baseline titers and sex. Dose level and adjuvant were categorical variables, and placebo recipients were excluded from the analysis. The full model included a dose level by adjuvant interaction term. A series of reduced models were fitted to test for no interaction between dose level and adjuvant, no effect of adjuvant, and no effect of dose level. The likelihood ratio and Wald tests were used to determine significance (nominal *P* value: ≤.05); *P* values of <.05 indicated that results may warrant further study. No adjustments for multiple testing were made. The effect of each dose level and comparison between dose levels were further assessed based on the selected model.

All analyses were conducted using SAS software version 9.4 or higher.

## RESULTS

### Participants

**Figure 1.**
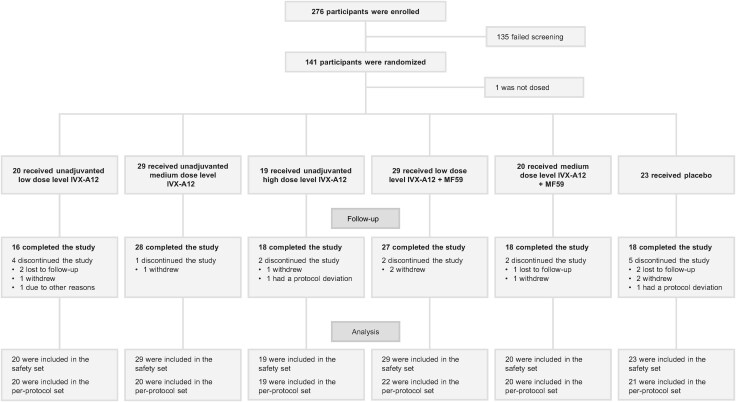
Participant disposition. Flowchart showing: the number of participants enrolled, randomized, and vaccinated with IVX-A12 at varying dose levels ± the adjuvant MF59^®^, or placebo; and the number of participants included in the safety and per-protocol sets. Exclusions from the per-protocol set are reported in the Supplementary Results. IVX-A12 dose levels were defined as follows: low, 75 µg respiratory syncytial virus (RSV)/75 µg human metapneumovirus (hMPV); medium, 75 µg RSV/150 µg hMPV; high, 75 µg RSV/225 µg hMPV.

A total of 276 participants were enrolled into 3 consecutive trial cohorts between September 2022 and January 2023. After screening, 141 were randomized and 140 received IVX-A12, with or without adjuvant, or placebo (Figure 1). All 140 vaccine recipients were included in the safety set, and of these, 122 were included in the per-protocol set. Exclusions from the per-protocol set are reported in the Supplementary Results. All follow-up of participants was completed by 24 January 2024.

Participant demographics and clinical characteristics were balanced between treatment groups and analysis sets (Table 1). In the safety set, median age was 65.0 years (range, 60–75 years) and mean body mass index was 28.1 kg/m^2^ (standard deviation, 3.7 kg/m^2^); the majority of participants were female (55.0% [n = 77/140]) and White (85.0% [n = 119/140]).

**Table 1.** Participant Demographics and Clinical Characteristics in Key Analysis Populations at Baseline

Characteristic	Unadjuvanted IVX-A12			IVX-A12 + MF59^®^		IVX-A12 Total	Placebo	Total
	Low	Medium	High	Low	Medium			
Safety set	n = 20	n = 29	n = 19	n = 29	n = 20	n = 117	n = 23	N = 140
Age, y								
Median (range)	67.0 (60–75)	66.0 (60–73)	64.0 (60–73)	64.0 (60–74)	63.0 (60–71)	64.0 (60–75)	65.0 (60–74)	65.0 (60–75)
Sex, No. (%)								
Male	11 (55.0)	9 (31.0)	8 (42.1)	14 (48.3)	11 (55.0)	53 (45.3)	10 (43.5)	63 (45.0)
Female	9 (45.0)	20 (69.0)	11 (57.9)	15 (51.7)	9 (45.0)	64 (54.7)	13 (56.5)	77 (55.0)
Race, No. (%)								
White	16 (80.0)	24 (82.8)	18 (94.7)	26 (89.7)	16 (80.0)	100 (85.5)	19 (82.6)	119 (85.0)
Black/African American	3 (15.0)	4 (13.8)	1 (5.3)	3 (10.3)	3 (15.0)	14 (12.0)	2 (8.7)	16 (11.4)
American Indian/Alaska Native	1 (5.0)	0	0	0	0	1 (0.9)	1 (4.3)	2 (1.4)
Native Hawaiian/Pacific Islander	0	1 (3.4)	0	0	0	1 (0.9)	0	1 (0.7)
Asian	0	0	0	0	0	0	0	0
Other	0	0	0	0	1 (5.0)	1 (0.9)	1 (4.3)	2 (1.4)
BMI, kg/m^2^								
Mean (SD)	27.8 (3.56)	27.9 (3.66)	26.5 (3.63)	28.3 (3.43)	29.1 (4.34)	28.0 (3.73)	28.9 (3.44)	28.1 (3.69)
Per-protocol set	n = 20	n = 20	n = 19	n = 22	n = 20	n = 101	n = 21	N = 122
Age, y								
Median (range)	67.0 (60–75)	66.0 (60–72)	64.0 (60–73)	64.5 (60–74)	63.0 (60–71)	64.0 (60–75)	65.0 (60–74)	65.0 (60–75)
Sex, No. (%)								
Male	11 (55.0)	8 (40.0)	8 (42.1)	10 (45.5)	11 (55.0)	48 (47.5)	9 (42.9)	57 (46.7)
Female	9 (45.0)	12 (60.0)	11 (57.9)	12 (54.5)	9 (45.0)	53 (52.5)	12 (57.1)	65 (53.3)
Race, No. (%)								
White	16 (80.0)	19 (95.0)	18 (94.7)	22 (100.0)	16 (80.0)	91 (90.1)	17 (81.0)	108 (88.5)
Black/African American	3 (15.0)	0	1 (5.3)	0	3 (15.0)	7 (6.9)	2 (9.5)	9 (7.4)
American Indian/Alaska Native	1 (5.0)	0	0	0	0	1 (1.0)	1 (4.8)	2 (1.6)
Native Hawaiian/Pacific Islander	0	1 (5.0)	0	0	0	1 (1.0)	0	1 (0.8)
Asian	0	0	0	0	0	0	0	0
Other	0	0	0	0	1 (5.0)	1 (1.0)	1 (4.8)	2 (1.6)
BMI, kg/m^2^								
Mean (SD)	27.8 (3.56)	27.3 (3.61)	26.5 (3.63)	28.4 (3.41)	29.1 (4.34)	27.8 (3.76)	29.4 (3.14)	28.1 (3.69)

The safety set included all participants who received IVX-A12 or placebo, per actual trial vaccination received. The per-protocol set included all participants who received IVX-A12 or placebo, per randomization, and had no major protocol deviations on or before day 28; exclusions from the per-protocol set are reported in the Supplementary Results. IVX-A12 dose levels were defined as follows: low, 75 µg RSV/75 µg hMPV; medium, 75 µg RSV/150 µg hMPV; high, 75 µg RSV/225 µg hMPV. Only 1 participant (who received medium dose level IVX-A12 + MF59^®^) did not have a history of medical disorders or procedures.

Abbreviations: BMI, body mass index; SD, standard deviation.

### Safety

**Figure 2.**
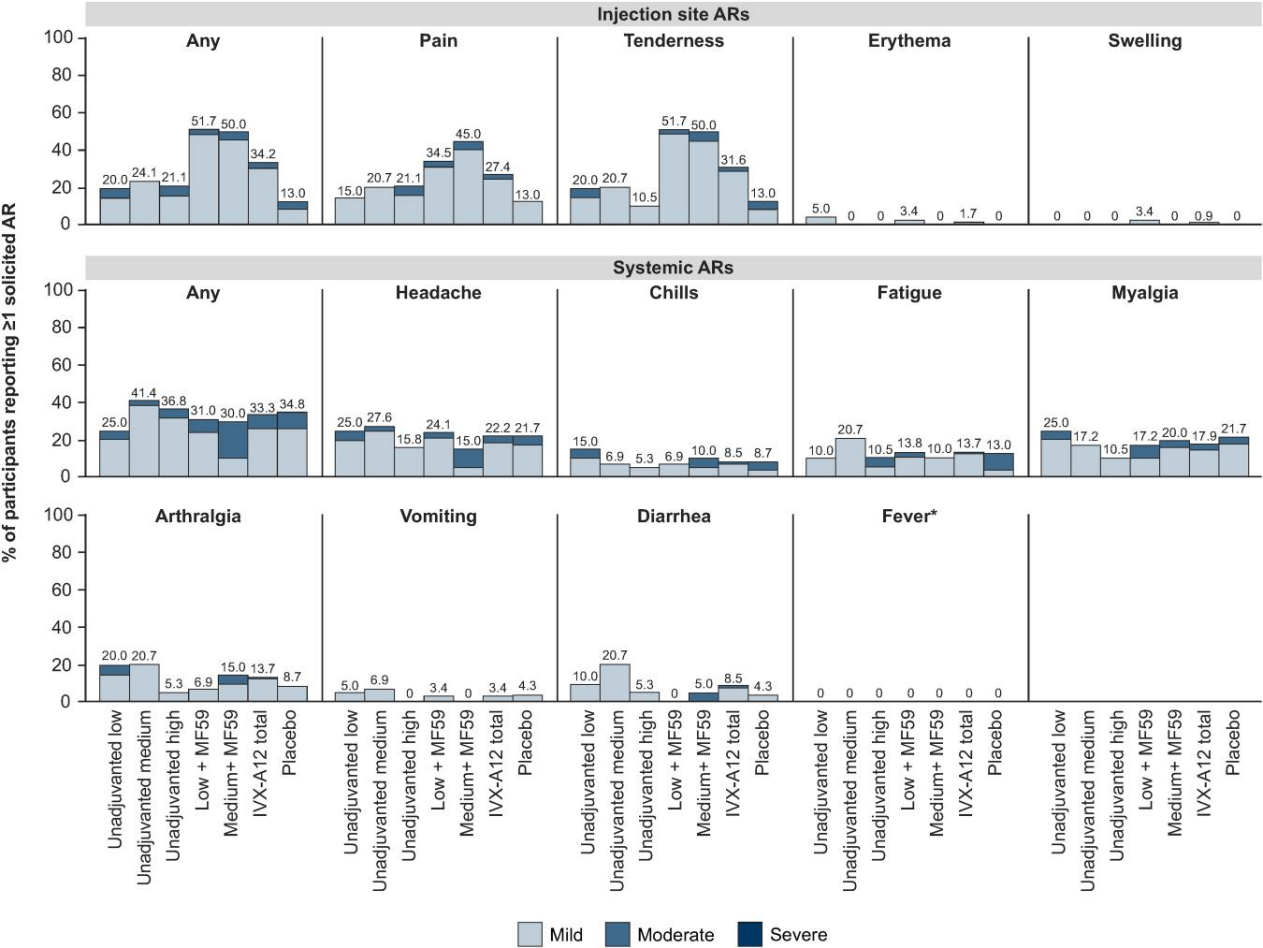
Injection site and systemic solicited adverse reactions (ARs) within 7 days after vaccination by severity. In the safety population (N = 140), the total numbers of participants per group were as follows: unadjuvanted low, n = 20; unadjuvanted medium, n = 29; unadjuvanted high, n = 19; low + MF59^®^, n = 29; medium + MF59^®^, n = 20; IVX-A12 total, n = 117; placebo, n = 23. Participants who reported multiple events of the same AR are counted once for that AR at the maximum severity reported. Severity was graded by the investigator on a 4-point scale: mild (grade 1), moderate (grade 2), severe (grade 3), or potentially life-threatening (grade 4). IVX-A12 dose levels were defined as follows: low, 75 µg respiratory syncytial virus (RSV)/75 µg human metapneumovirus (hMPV); medium, 75 µg RSV/150 µg hMPV; high, 75 µg RSV/225 µg hMPV. *Fever was defined as body temperature ≥38°C (≥100.4°F), regardless of the method used.

Overall, 47.0% (n = 55/117) of participants who received IVX-A12 and 34.8% (n = 8/23) of participants who received placebo reported ≥1 solicited AR within 7 days of vaccination; all were transient with mild to moderate severity. A summary of injection site and systemic ARs by severity is presented in Figure 2.

Injection site ARs were reported by 34.2% (n = 40/117) of participants who received IVX-A12 and 13.0% (n = 3/23) who received placebo, with tenderness (31.6% [n = 37/117]) and pain (27.4% [n = 32/117]) reported most frequently by IVX-A12 recipients. Incidence of injection site ARs did not increase with increasing hMPV VLP dose level; however, incidence was numerically lower in participants who received unadjuvanted compared with adjuvanted IVX-A12.

Systemic ARs were reported by 33.3% (n = 39/117) of participants who received IVX-A12 and 34.8% (n = 8/23) who received placebo; incidence was similar across IVX-A12 dose levels and regardless of adjuvant use. The most frequently reported systemic ARs in IVX-A12 recipients were headache (22.2% [n = 26/117]) and myalgia (17.9% [n = 21/117]).

A summary of AEs is presented in Supplementary Table 1. Unsolicited AEs through day 28 were reported by 26.5% (n = 31/117) of participants who received IVX-A12 and 26.1% (n = 6/23) who received placebo; the majority were mild or moderate. Three IVX-A12 recipients (2.6%) reported severe AEs: 2 events of increased blood pressure and 1 of hypertension, all of which were considered unrelated to vaccination. MAAEs through day 365 were reported by 39.3% (n = 46/117) of IVX-A12 recipients and 34.8% (n = 8/23) of placebo recipients; none were considered related to vaccination. The most frequently reported system organ class for unsolicited AEs and MAAEs was Infections and Infestations. SAEs through day 365 were reported by 3.4% (n = 4/117) and 4.3% (n = 1/23) of IVX-A12 and placebo recipients, respectively, with none considered related to vaccination. SAEs by preferred term are listed in Supplementary Table 1. There were no RSV or hMPV infections, AESIs (moderate to severe LRTIs), AEs leading to trial discontinuation, or deaths through day 365.

### Immunogenicity

**Figure 3.**
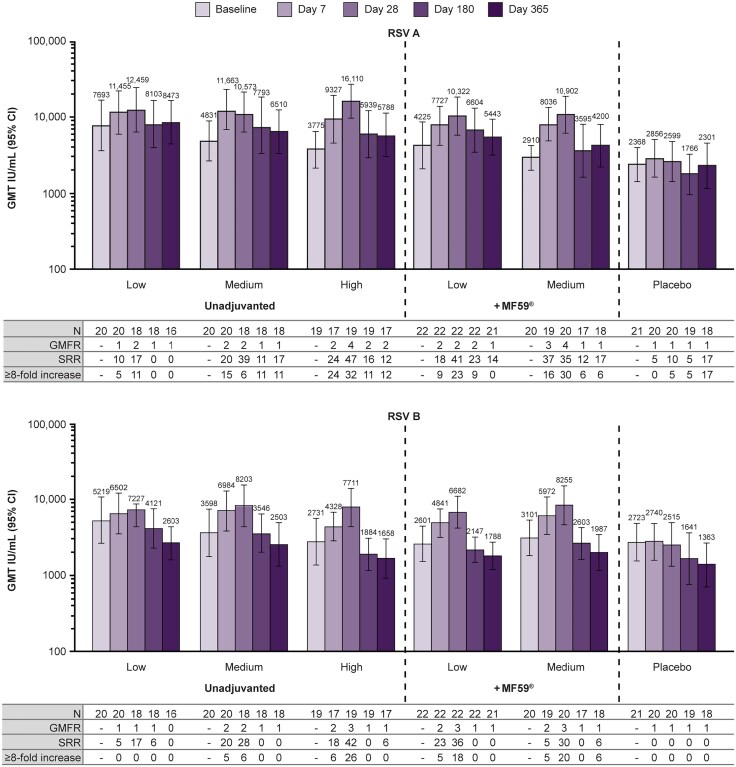

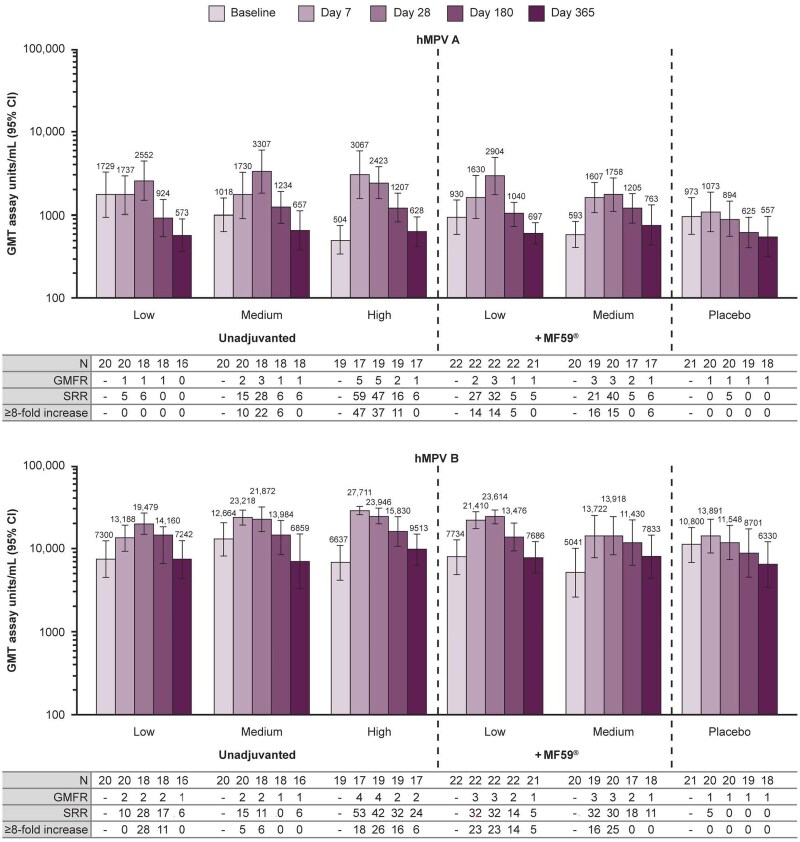
Respiratory syncytial virus (RSV) A/B– and human metapneumovirus (hMPV) A/B–specific neutralizing antibody (nAb) titers up to day 365. nAb titers were assessed prior to vaccination on day 0 (baseline), and postvaccination on days 7, 28, 180, and 365 in the per-protocol set (N = 122); data for n = 7 participants were excluded at specific timepoints, as summarized in the Supplementary Results. Geometric mean fold rises, percentages of participants with a ≥4-fold increase in nAb titers (seroresponse rate), and percentages of participants with a ≥8-fold increase in nAb titers at each timepoint were calculated from baseline. IVX-A12 dose levels were defined as follows: low, 75 µg RSV/75 µg hMPV; medium, 75 µg RSV/150 µg hMPV; high, 75 µg RSV/225 µg hMPV. Abbreviations: CI, confidence interval; GMT, geometric mean titer; IU, international units; SRR, seroresponse rate.

RSV A/B- and hMPV A/B-specific nAb GMTs and GMFRs through day 365 are summarized in Figure 3. High baseline titers of RSV-specific nAbs were observed, particularly in participants enrolled to the earliest cohort receiving unadjuvanted low dose level IVX-A12. IVX-A12 induced a 2- to 4-fold increase in RSV A nAb titers and a 1- to 3-fold increase in RSV B nAb titers from baseline to day 28, compared with a 1-fold increase induced by placebo. IVX-A12 also induced a 1- to 5-fold increase in hMPV A nAb titers and a 2- to 4-fold increase in hMPV B nAb titers from baseline to day 28, compared with a 1-fold increase induced by placebo. In IVX-A12 recipients, RSV- and hMPV-specific nAb GMTs increased from baseline to day 7, were measured highest at day 28, then gradually declined but remained above or around baseline levels through day 365. nAb GMTs in IVX-A12 recipients remained higher than those in placebo recipients at all timepoints through day 365, across all 4 viral subgroups. SRRs and percentages of participants with a ≥8-fold increase in nAb titers are detailed in Figure 3. SRRs were observed to peak at day 28 across viral subgroups, and a higher proportion of IVX-A12 recipients were seroresponders at day 28 compared with placebo recipients.

RSV- and hMPV-specific nAb titers were also analyzed by baseline tertile to assess whether or not the high baseline RSV-specific nAb titers confounded the immunogenicity results (Supplementary Figure 2; Supplementary Table 2). GMFRs were highest in participants with the lowest baseline nAb titers (first tertile) and lowest in participants with the highest baseline nAb titers (third tertile). For participants with nAb titers in the first tertile, IVX-A12 induced a 2- to 11-fold increase in RSV A nAb titers and a 3- to 7-fold increase in RSV B nAb titers from baseline to day 28, compared with a 1- to 2-fold increase induced by placebo. Additionally, IVX-A12 induced a 4- to 9-fold increase in hMPV A nAb titers and a 6- to 9-fold increase in hMPV B nAb titers from baseline to day 28, compared with a 1-fold increase induced by placebo. GMTs and GMFRs for participants in the second and third tertiles are reported in Supplementary Table 2.

**Figure 4.**
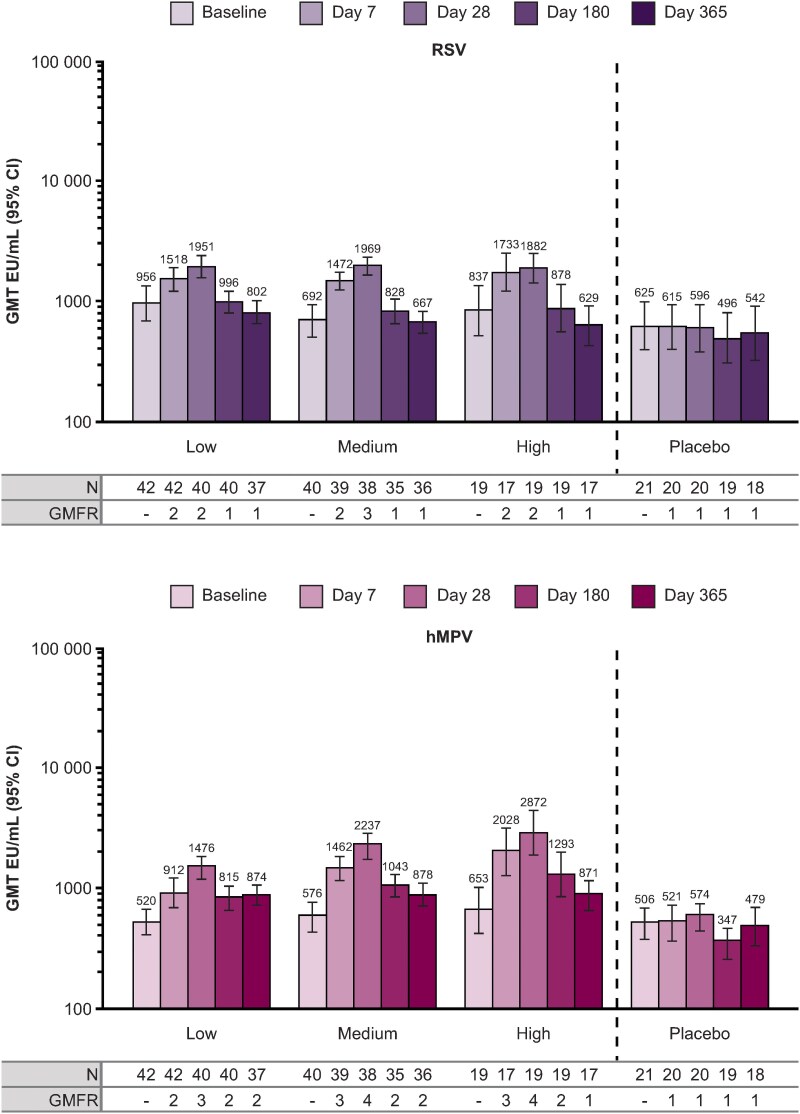
Respiratory syncytial virus (RSV)– and human metapneumovirus (hMPV)–specific prefusion conformation of the fusion protein (pre-F) immunoglobulin G (IgG) titers by pooled dose level up to day 365. Pre-F IgG titers were assessed prior to vaccination on day 0 (baseline), and postvaccination on days 7, 28, 180, and 365 in the per-protocol set (N = 122); data for n = 7 participants were excluded at specific timepoints, as summarized in the Supplementary Results. Geometric mean fold rises at each timepoint were calculated from baseline. IVX-A12 dose levels were defined as follows: low, 75 µg RSV/75 µg hMPV; medium, 75 µg RSV/150 µg hMPV; high, 75 µg RSV/225 µg hMPV. Abbreviations: CI, confidence interval; EU, enzyme-linked immunosorbent assay units; GMT, geometric mean titer.

RSV- and hMPV-specific pre-F IgG GMTs through day 365 by pooled IVX-A12 dose level (independent of adjuvant) are shown in Figure 4. IVX-A12 induced a 2- to 3-fold increase in RSV pre-F IgG titers and a 3- to 4-fold increase in hMPV pre-F IgG titers from baseline to day 28, compared with a 1-fold increase induced by placebo. In IVX-A12 recipients, GMTs increased from baseline to day 28, then returned to near-baseline levels by day 180 for RSV-specific pre-F IgG and by day 365 for hMPV-specific pre-F IgG.

Linear regression analyses showed that inclusion of adjuvant had no effect on RSV- or hMPV-specific antibody levels at day 28. Dose level, however, did have an effect on day 28 hMPV B–specific nAb responses (likelihood ratio test: *P* = .018) and hMPV-specific pre-F IgG responses (likelihood ratio test: *P* = .001; Supplementary Table 3). The effect of the high dose level was different from the medium dose level on hMPV B–specific nAb responses (Wald test: *P* = .026), while the effect of the medium dose level was not different from the low dose level (Wald test: *P* = .404); adjusted GMFRs were 3, 2, and 3 for the low, medium, and high pooled dose levels, respectively. For hMPV-specific pre-F IgG responses, adjusted GMFRs were 3, 4, and 5 for the low, medium, and high pooled dose levels, respectively. Additionally, dose level had an effect on RSV A–specific nAb responses (likelihood ratio test: *P* = .048) at day 28. The effect of the high dose level was different from the low dose level on RSV A–specific nAb responses (Wald test: *P* = .046); adjusted GMFRs for the high and low pooled dose levels were 4 and 2, respectively.

GMR estimates of fold rise in pre-F IgG titers from baseline over fold rise in nAb titers from baseline were <1 at most timepoints, indicating potential selective induction of nAb responses; however, confidence intervals were generally wide and overlapped with 1 (Supplementary Table 4).

VLP core-specific IgG GMTs and GMFRs through day 365 are displayed in Supplementary Figure 3. Overall, VLP core-specific IgG responses were low (near the lower limit of quantification for the assay) and remained stable or declined through day 365. GMTs and GMFRs were slightly lower in participants who received unadjuvanted versus adjuvanted IVX-A12.

## DISCUSSION

Awareness of the significant impact of several respiratory viruses in older adults is increasing [1, 8] and necessitates a combination approach to vaccination to expand protection to cover important viruses that cause LRTDs, such as RSV and hMPV, while facilitating vaccine uptake [22]. This first-in-human trial of an RSV/hMPV combination VLP vaccine demonstrated that IVX-A12 was well-tolerated in adults 60–75 years of age, had an acceptable safety profile, and elicited humoral immune responses against RSV and hMPV that were maintained above or around baseline for up to 1 year.

Consistent with phase 1 safety analyses of the licensed protein-based RSV vaccines, RSVpreF3 and RSVpreF [23, 24], transient mild to moderate reactogenicity was observed within 7 days of IVX-A12 vaccination. Rates of solicited injection site reactions were higher in IVX-A12 recipients (34%) compared with placebo recipients (13%), while rates of solicited systemic reactions were similar (33% and 35%, respectively). These results are comparable with the previous phase 1 analysis of the RSV component of IVX-A12, in which 32% of RSV VLP recipients had solicited injection site reactions, and rates of solicited systemic reactions were similar for the RSV VLP (14%) and placebo (16%) groups [20]. Although no IVX-A12 dose-response relationship was observed for injection site or systemic reactogenicity, rates of injection site reactions were slightly higher for IVX-A12 formulations containing adjuvant versus those without. Additionally, there were no vaccine-related moderate/severe AEs, SAEs, or MAAEs, and no AESIs, AEs leading to trial discontinuation, or deaths. These results indicate that IVX-A12 was well tolerated with no unexpected safety concerns in this healthy population of adults 60–75 years of age, supporting expanded clinical evaluation of this protein-based VLP vaccine.

A single dose of IVX-A12 boosted preexisting RSV-specific nAb and pre-F IgG titers at all IVX-A12 dose levels and formulations analyzed. While no correlate of protection for RSV has been formally established, RSV nAb levels in serum are associated with a reduced risk of RSV hospitalization and thus are frequently used to assess RSV interventions [10, 25, 26]. Phase 3 trials of the licensed RSVpreF3 and mRNA-1345 vaccines reported robust increases in RSV nAb titers at 1 month postvaccination, ranging from 8- to 11-fold for RSV A and 5- to 8-fold for RSV B [27, 28]. One hypothesis is that the relatively low fold rises seen in this trial were a result of the high levels of baseline immunity against RSV. During the 2022–2023 RSV season, there was an atypical early and severe RSV epidemic in Florida (where the majority [69%] of participants were enrolled) [29, 30]; RSV infections reached a peak in late July 2022 and dosing for this trial began in late September 2022 [29, 30]. Only individuals with self-reported, laboratory-confirmed severe RSV infection were excluded. Therefore, participants enrolled to the earlier cohorts may have had undetected or unconfirmed RSV infection just before trial entry, leading to the observed high levels of RSV nAb titers at baseline, and a potential underestimation of IVX-A12 immunogenicity. Further analyses indicated that the largest increases in nAb responses were found in participants with antibody titers in the lowest baseline tertile.

All IVX-A12 dose levels and formulations boosted preexisting hMPV-specific nAb and pre-F IgG titers. Although protective immune effector mechanisms for hMPV have not yet been defined, evidence suggests that hMPV nAb levels in serum are associated with protection against hMPV infection [31]. Convalescent sera following natural hMPV infection show a 2- to 3-fold increase in hMPV nAb titers [31], which are comparable with the increases in hMPV A/B nAb titers observed at 1 month after IVX-A12 vaccination; however, the protection conferred by natural infection is short-lived [11].

Encouragingly, both RSV A/B and hMPV A/B nAb responses elicited by IVX-A12 were maintained above or around baseline and exceeded those in placebo recipients through 1 year, while antibody responses to the VLP core remained low for the duration of the study. The durability of IVX-A12–induced humoral immunity is important, as the RSV season in the Northern Hemisphere typically begins in October and peaks during the winter months, while the hMPV season begins later and peaks from late winter to spring [32]. These data indicate that humoral responses following a single dose of IVX-A12 may have the potential to persist for the combined RSV and hMPV seasons.

Increasing amounts of hMPV VLP did not increase day 28 hMPV A nAb titers. However, hMPV-specific pre-F IgG levels did increase with hMPV dose level. Increasing hMPV dose level also affected hMPV B nAb titers, but without a clear dose-response curve as measured by adjusted GMFRs. Interpretation of the results for hMPV B nAbs was confounded by almost half of participants having baseline or day 28 hMPV B nAb titers at the upper limit of quantification for the assay. Importantly, the increasing hMPV dose levels did not interfere with induction of RSV nAbs. The impact of different IVX-A12 dose levels and adjuvant are being further investigated in phase 2 trials (NCT05903183).

Taken together, these safety and immunogenicity data demonstrate the potential of this combination, protein-based VLP vaccine against RSV- and hMPV-associated LRTD. While an increasing number of vaccines are recommended to protect older adults against viruses that cause LRTDs, including RSV, influenza, and severe acute respiratory syndrome coronavirus 2 [33], there remains an unmet need to protect against hMPV LRTD. Therefore, combination vaccination represents a rational approach to provide additional protection against hMPV, without impacting recipient uptake or efficiency for healthcare providers [22].

In addition to the impact of high baseline RSV immunity from recent exposure, the other limitations of this analysis are inherent to first-in-human trials and include the small population size and the eligibility criteria that limited the population to relatively healthy adults 60–75 years of age. Individuals with stable, well-controlled, chronic conditions were included but are not representative of the older adult population at highest risk for severe RSV and hMPV LRTD [1, 8].

Overall, a single dose of IVX-A12 was well-tolerated and induced RSV- and hMPV-specific antibody responses in adults 60–75 years of age. These results support the continued clinical evaluation of IVX-A12 for protection against RSV- and hMPV-associated LRTD and highlight the potential of this combinatorial protein-based VLP vaccine approach.

## Supplementary Material

ofaf160_Supplementary_Data

## Supplementary Data

Supplementary materials are available at *Open Forum Infectious Diseases* online. Consisting of data provided by the authors to benefit the reader, the posted materials are not copyedited and are the sole responsibility of the authors, so questions or comments should be addressed to the corresponding author.

## Notes

***Acknowledgments.*** The trial (with protocol ICVX-12-101) was designed and undertaken by the sponsor, and clinical safety monitoring was provided by Syneos Health (Morrisville, North Carolina, USA). All immunogenicity assays were performed at Cerba Research (Rotterdam, the Netherlands). The authors thank all participants and site personnel involved in this clinical trial; the scientific advisers Ralf Clemens, Florian Schödel, and John Shriver for their input; the members of the independent safety monitoring committee (Fred Zepp, MD [Chairman], Stephen J. Thomas, MD, and Frank Rockhold, PhD); Marta Murreddu, Danny Duijsings, and Joëlle Vink (Cerba Research) for overseeing clinical sample testing and data management coordination; and Syneos Health for expert data management, statistical analyses, and site monitoring activities, in particular, Kristin Ritter, Myra Miller, Lisa Serrichio, Miriam Sarabia, Laura J. Stephens, Morgan Kearny, and Laura Spurchise. The authors also thank Amy Kinney (consultant) for help in coordinating operational activities; Cheryl Pikora (consultant) for additional clinical safety monitoring assistance; Xiaohua Sheng (Dubu Research) for additional statistical support; Dustin Leslie (Icosavax) for project management coordination; Thalia Nguyen (Icosavax) for ensuring adequate vaccine, adjuvant, and placebo supplies; and Ajoke Sobanjo-ter Meulen for provision of epidemiology data in support of hMPV vaccine evaluation. Medical writing support was provided by Emily Smyth of Ashfield MedComms, an Inizio company, in accordance with Good Publication Practice 2022 guidelines (https://www.ismpp.org/gpp-2022), and funded by AstraZeneca.

***Author contributions.*** Conceptualization or trial design: J. P., J. W., M. C., N. H., and N. K. Data acquisition, analysis, or interpretation: A. E. E., C. S., E. M. A., J. P., M. C., N. H., N. K., N. S.-C., and W. R. Writing or revising the work: All authors. All authors provided final approval of the version to be published and agree to be accountable for all aspects of the work.

***Data availability.*** Data underlying the findings described in this manuscript may be obtained in accordance with AstraZeneca's data sharing policy described at https://www.astrazenecaclinicaltrials.com/our-transparency-commitments/. Data for studies directly listed on Vivli can be requested through Vivli at www.vivli.org. Data for studies not listed on Vivli can be requested through Vivli at https://vivli.org/members/enquiries-about-studies-not-listed-on-the-vivli-platform/. The AstraZeneca Vivli member page outlining further details is also available at https://vivli.org/ourmember/astrazeneca/.

***Financial support.*** This work was supported by Icosavax, a member of the AstraZeneca group.
