# Supplementary material for: A Randomized Phase 1 Clinical Trial of a Respiratory Syncytial Virus and Human Metapneumovirus Combination Protein-Based Virus-like Particle Vaccine in Adults 60–75 Years of Age
Source: Open Forum Infect Dis. 2025 Mar 13;12(4):ofaf160. doi: 10.1093/ofid/ofaf160 (PMC11977332; doi:10.1093/ofid/ofaf160)
Supplement: ofaf160_Supplementary_Data [file ofaf160_supplementary_data.docx]

**A randomized Phase 1 clinical trial of a respiratory syncytial virus and human metapneumovirus combination protein-based virus-like particle vaccine in adults 60 to 75 years of age**

Craig Shapiro, Nelia Sánchez-Crespo, Max Ciarlet, Nicholas Hourguettes, Judy Wen, Wasima Rida, Jennifer Price, April E. Engram, Elizabeth M. Adams, and Niranjan Kanesa-thasan

# Supplementary materials

| **Contents** | **Page** |
| --- | --- |
| **Supplementary Methods** | **2** |
| Stopping rules | 2 |
| Eligibility criteria | 2 |
| Clinical event of special interest | 5 |
| Severity grading for solicited ARs and unsolicited AEs | 5 |
| Serological assessments | 7 |
| **Supplementary Results** | **9** |
| Participant exclusions from the per-protocol set | 9 |
| **Supplementary Tables** | **10** |
| Table S1. Summary of unsolicited AEs, SAEs, MAAEs, AESIs, and AEs leading to trial discontinuation | 10 |
| Table S2. RSV- and hMPV-specific nAb GMTs and GMFRs by baseline nAb titer tertile up to Day 365 | 12 |
| Table S3. Linear regression for IVX-A12 formulation selection and dose level guidance | 25 |
| Table S4. GMRs to assess selectivity for nAb or pre-F IgG responses against RSV and hMPV | 27 |
| **Supplementary Figures** | **29** |
| Figure S1. Study design | 29 |
| Figure S2. RSV- and hMPV-specific nAb titers in participants with baseline nAb titers in the first tertile up to Day 365 | 30 |
| Figure S3. VLP core-specific IgG titers up to Day 365 | 32 |
| **Supplementary References** | **33** |

# Supplementary Methods

## Stopping rules

In a trial participant:

- Any death occurring during the trial
- Any vaccine-related serious adverse event (SAE) during the trial
- Any life-threatening (Grade 4) vaccine-related adverse event (AE) during the trial that needs medical intervention, including: ulceration, abscess or necrosis at the injection site; laryngospasm, bronchospasm, or anaphylaxis within 24 hours after administration of vaccine
- An allergic or hypersensitivity reaction such as fever >40°C or generalized urticaria (defined as occurring at three or more body parts) within 72 hours after administration of vaccine

In two or more participants in a single treatment group in any of the three trial cohorts:

- The same severe (Grade 3) AE within the first 7 days following vaccination that persisted for at least 48 hours and cannot be clearly attributed to another cause, including: severe (Grade 3) injection site solicited adverse reaction (AR; excluding measured grades of erythema and swelling alone) or systemic solicited AR; severe (Grade 3) vaccine-related unsolicited AE during the trial; severe (Grade 3) vaccine-related vital sign(s) abnormality; severe (Grade 3) vaccine-related clinical laboratory abnormality

## Eligibility criteria

Inclusion criteria – each participant met the following criteria to be enrolled in the trial:

1. Healthy male or non-pregnant female adults 60 to 75 years of age at the time of vaccination
   1. Includes subjects with stable well-controlled chronic conditions such as hypertension without clinical exacerbation of their underlying disease within the previous 12 months
2. Subjects able to voluntarily give written informed consent and to comply with trial procedures including follow-up to approximately 12 months after dosing
3. Body mass index 17 to 35 kg/m^2^, inclusive, at screening
4. Screening laboratory values must be within the laboratory reference ranges or deemed not clinically significant if within Grade 1 severity on the toxicity scale

Exclusion criteria – participants meeting any of the following criteria were excluded from the trial:

1. Prior receipt of any investigational respiratory syncytial virus (RSV) or human metapneumovirus (hMPV) vaccine
2. Prior receipt of another investigational medicinal product (study drug, biologic, or device) not authorized for use in the United States (US) within the past year
3. Laboratory-confirmed severe RSV or hMPV infection within the past year prior to enrollment
4. Currently enrolled or plan to participate in another clinical trial with an investigational agent (including licensed or unlicensed vaccine, drug, biologic, device, blood product, or medication) to be received during the trial period
5. Presence of high-risk comorbidities for severe RSV or hMPV disease (significant cardiopulmonary disease, as described below):
   1. Chronic obstructive pulmonary disease, asthma requiring daily medications currently or any treatment of respiratory disease exacerbations in the last 5 years
   2. Congestive heart failure, cardiomyopathy, ischemic heart disease, or history of myocarditis or pericarditis as an adult
6. Older adults meeting frail elderly criteria (older persons with medical, nutritional, cognitive, emotional, or activity impairments, as defined by the trial site)
7. Acute or chronic progressive, unstable, or uncontrolled clinical conditions including:
   1. Ongoing malignancy or recent diagnosis of malignancy in the last 5 years excluding basal cell and squamous cell carcinoma of the skin, which are allowed
   2. An autoimmune disease, including hypothyroidism without a defined non-autoimmune cause, localized psoriasis or history of psoriasis
   3. Chronic renal or liver disease, including fatty liver
8. Acute illness with or without fever at the time of planned vaccination
9. History of hypersensitivity or serious adverse reactions to vaccines, such as anaphylaxis, Guillain-Barré, and angioedema, any known allergies to any component of the IVX-121 and/or IVX-241 vaccine, or hypersensitivity to latex
10. Abnormal function of the immune system resulting from clinical conditions including human immunodeficiency virus, chronic administration of systemic corticosteroids (oral/intravenous/intramuscular [IM] at a dosage equivalent of >20 mg prednisone in a period of more than 14 days), or administration of immunosuppressive chemotherapy, biologics, or radiotherapy within the past 3 months before trial randomization
11. Positive for human immunodeficiency virus, hepatitis B surface antigen, and hepatitis C virus antibody
12. Positive urine dipstick for protein and glucose (positive to trace protein is acceptable)
13. Clinical conditions representing a contraindication to IM injection, e.g. blood dyscrasias or significant disorders of coagulation
14. Receipt of immunoglobulins or any blood products within the past 3 months before trial randomization
15. Intention to donate blood or plasma during the trial
16. Pregnant (positive pregnancy test) or lactating female
17. Refusal to maintain contraceptive practices during the trial, and (for women of childbearing potential) to be screened for pregnancy at specified times during the trial
18. History of any neurological or neurodevelopmental disorders, e.g. severe migraines in the past 5 years, epilepsy, stroke, seizures in the last 3 years, encephalopathy, focal neurologic deficits, Guillain-Barré syndrome, encephalomyelitis, or transverse myelitis
19. Trial personnel as an immediate family or household member
20. Other clinical condition that, in the opinion of the investigator, might pose additional risk of injury to the subject, or interfere with the results of the trial or with the ability of the subject to participate in the trial
21. Receipt of licensed inactivated vaccines including influenza vaccine within 14 days prior to trial vaccine administration on Day 0, or with live virus vaccines within 30 days of Day 0
    1. Receipt of licensed vaccines is permitted after completion of the trial Day 28 visit
    2. Receipt of licensed coronavirus disease 2019 vaccines is permitted if dosing regimen completed within 21 days prior to trial vaccine administration on Day 0 or after completion of the Day 28 visit

There may be instances when individuals meet all entry criteria except one that relates to transient clinical circumstances (e.g. fever or recent use of excluded medication[s] or vaccine[s]). Under these circumstances, eligibility for enrollment may be considered if the appropriate window for delay has passed, inclusion/exclusion criteria have been rechecked, and if the subject is confirmed to be eligible.

## Clinical event of special interest (CESIs)

The only prespecified CESI in this trial was moderate (Grade 2) to severe (Grade 3) lower respiratory tract infection occurring after vaccination (Day 0) through to trial end (Day 365), defined as the presence of ≥3 of the following symptoms:

- Cough
- Wheezing (or worsening in baseline wheezing)
- New sputum production (or increase in baseline sputum production)
- New (or worsening) shortness of breath
- Fever (>38°C)
- Tachypnea (≥20 breaths per minute)

## Severity grading for solicited ARs and unsolicited AEs

Severity of solicited ARs was graded according to the following parameters:

| **Injection site solicited ARs** | |  |
| --- | --- | --- |
| Pain | Grade 1 | Mild: able to perform duties |
|  | Grade 2 | Moderate: interferes with duties |
|  | Grade 3 | Severe: prevents duties |
|  | Grade 4 | Emergency room (ER) visit or hospitalization |
| Tenderness | Grade 1 | Mild: discomfort to touch |
|  | Grade 2 | Moderate: discomfort with movement |
|  | Grade 3 | Severe: significant discomfort at rest |
|  | Grade 4 | ER visit or hospitalization |
| Erythema | Grade 1 | Mild: ≥25–≤50 mm |
|  | Grade 2 | Moderate: >50–≤100 mm |
|  | Grade 3 | Severe: >100 mm |
|  | Grade 4 | Necrosis or exfoliative dermatitis |
| Swelling | Grade 1 | Mild: ≥25–≤50 mm |
|  | Grade 2 | Moderate: >50–≤100 mm |
|  | Grade 3 | Severe: >100 mm |
|  | Grade 4 | Necrosis |
| **Systemic solicited ARs** | |  |
| Headache | Grade 1 | Mild: no interference with activity |
|  | Grade 2 | Moderate: some interference with activity |
|  | Grade 3 | Severe: prevents daily activity |
|  | Grade 4 | ER visit or hospitalization |
| Chills | Grade 1 | Mild: no interference with activity |
|  | Grade 2 | Moderate: some interference with activity |
|  | Grade 3 | Severe: prevents daily activity |
|  | Grade 4 | ER visit or hospitalization |
| Fatigue | Grade 1 | Mild: no interference with activity |
|  | Grade 2 | Moderate: some interference with activity |
|  | Grade 3 | Severe: prevents daily activity |
|  | Grade 4 | ER visit or hospitalization |
| Myalgia | Grade 1 | Mild: no interference with activity |
|  | Grade 2 | Moderate: some interference with activity |
|  | Grade 3 | Severe: prevents daily activity |
|  | Grade 4 | ER visit or hospitalization |
| Arthralgia | Grade 1 | Mild: no interference with activity |
|  | Grade 2 | Moderate: some interference with activity |
|  | Grade 3 | Severe: prevents daily activity |
|  | Grade 4 | ER visit or hospitalization |
| Vomiting | Grade 1 | Mild: no interference with activity or 1–2 episodes/24 hours |
|  | Grade 2 | Moderate: some interference with activity or >2 episodes/24 hours |
|  | Grade 3 | Severe: prevents daily activity, requires outpatient intravenous hydration |
|  | Grade 4 | ER visit or hospitalization for hypotensive shock |
| Diarrhea | Grade 1 | Mild: 2–3 loose stools or <400 g/24 hours |
|  | Grade 2 | Moderate: 4–5 loose stools or 400–800 g/24 hours |
|  | Grade 3 | Severe: ≥6 watery stools or >800 g/24 hours or requires outpatient intravenous hydration |
|  | Grade 4 | ER visit or hospitalization for hypotensive shock |
| Fever* | Grade 1 | Mild: 38.0–38.4 °C/100.4–101.1 °F |
|  | Grade 2 | Moderate: 38.5–38.9 °C / 101.2–102.0 °F |
|  | Grade 3 | Severe: 39.0–40.0 °C / 102.1–104.0 °F |
|  | Grade 4 | >40.0 °C/>104.0 °F |

*Fever was defined as body temperature ≥38°C/100.4°F, regardless of the method used.

Severity of unsolicited AEs was graded by the Investigator according to the following US Food and Drug Administration grading scale:^1^

| Mild | Grade 1 | Awareness of symptoms that are easily tolerated, causing minimal discomfort and not interfering with everyday activities. Relieved with or without symptomatic treatment |  |
| --- | --- | --- | --- |
| Moderate | Grade 2 | Sufficient discomfort is present to cause interference with normal activity. Only partially relieved with symptomatic treatment or requires repeated use of non-narcotic pain reliever >24 hours. | |
| Severe | Grade 3 | Extreme distress, causing significant impairment of functioning or incapacitation. Prevents normal everyday activities. Not relieved with symptomatic treatment or requires use of narcotic pain reliever. | |
| Potentially  life-threatening | Grade 4 | Inability to perform basic self-care functions with intervention indicated to prevent permanent impairment, persistent disability, or death. | |

## Serological assessments

RSV A/B- and hMPV A/B-specific neutralizing antibody (nAb) titers in sera were assessed using virus neutralization assays. Briefly, a constant concentration of live virus (RSV A2; RSV B 18537; hMPV A1 NL/1/00; or hMPV B1 NL/1/99) was preincubated with serial dilutions of serum and was subsequently inoculated onto Hep-2 (RSV) or Vero (hMPV) target cells. nAb titers were measured through the inhibition of virus propagation in target cells by determining the serum dilution corresponding to a selected reduction in spot count (e.g. 50% for IC50). The lower limit of quantification (LLoQ) for nAb titers against RSV A was 9.4 international units (IU)/mL, for RSV B was 8.0 IU/mL and for hMPV A/B was 4.0 log_2_ assay units/mL. All analyses were performed at Cerba Research (Rotterdam, NL) using validated assays.

Titers of immunoglobulin G (IgG) specific to the RSV and hMPV prefusion proteins (pre-F), as well as the virus-like particle (VLP) core, were evaluated using enzyme-linked immunosorbent assays (ELISAs). Individual ELISA plates were coated with each antigen (RSV pre-F, hMPV pre-F, or VLP core protein). After blocking unknown samples, standard curve and control samples were added to the ELISA plates and incubated. After washing, horseradish peroxidase-conjugated anti-human IgG detection antibody was added, followed by incubation and subsequent washing. The amount of antigen-specific IgG binding was subsequently quantified by measuring the optical density after addition of tetramethylbenzidine substrate. Using the standard curve as a reference with assigned concentrations per level, the antigen-binding IgG concentration in samples was computed and reported as ELISA Units (EU)/mL. The LLoQ for IgG titers against RSV pre-F was 0.8 ELISA units (EU)/mL, for hMPV pre-F was 6.4 EU/mL, and for VLP core was 4.3 EU/mL. All analyses were performed at Cerba Research (Rotterdam, NL) using qualified assays.

# Supplementary Results

## Participant exclusions from the per-protocol set

Participants without a serum sample collected at Day 0 (n = 17) were replaced. These participants were followed for safety but were not included in the per-protocol set. Overall, n = 18 participants had major protocol deviations affecting immunogenicity outcomes for Day 28 and were excluded from the per-protocol set at all timepoints, including the n = 17 who did not have Day 0 serum samples and n = 1 who used systemic corticosteroids for more than 14 days around Day 28. Additionally, data for n = 7 participants in the per-protocol set were excluded at specific timepoints; n = 3 participants were excluded at Day 7 and n = 2 at Day 28 for immunogenicity samples outside the window, n = 1 participant was excluded at Days 180 and 365 for receiving an ongoing course of prednisone, and n = 1 participant was excluded at Day 365 for receiving another RSV vaccine.

# Supplementary Tables

## Table S1. Summary of unsolicited AEs, SAEs, MAAEs, AESIs, and AEs leading to trial discontinuation

Unsolicited AEs were collected through Day 28, and SAEs, MAAEs, AESIs, and AEs leading to trial discontinuation were collected through Day 365 in the safety population (N = 140). All AEs were unsolicited unless categorized as solicited ARs and reported within 7 days of vaccination. AEs were assessed for causality (related or not related to the trial investigational product) and severity (mild [Grade 1], moderate [Grade 2], severe [Grade 3], or life-threatening [Grade 4]) by the investigator. IVX-A12 dose levels were defined as follows: low = 75 µg RSV/75 µg hMPV; medium = 75 µg RSV/150 µg hMPV; high = 75 µg RSV/225 µg hMPV.

AE, adverse event; AR, adverse reaction; AESI, AE of special interest; hMPV, human metapneumovirus; MAAE, medically attended AE; RSV, respiratory syncytial virus; SAE, serious AE.

|  | **Unadjuvanted** | | |  | **+ MF59^®^** | |  |  |  |
| --- | --- | --- | --- | --- | --- | --- | --- | --- | --- |
| **Unsolicited AEs, n (%)** | **Low**  n = 20 | **Medium**  n = 29 | **High**  n = 19 |  | **Low**  n = 29 | **Medium**  n = 20 | **IVX-A12 total**  n = 117 | **Placebo**  n = 23 | **Total**  N = 140 |
| **Any AE** | 5 (25.0) | 11 (37.9) | 4 (21.1) |  | 7 (24.1) | 4 (20.0) | 31 (26.5) | 6 (26.1) | 37 (26.4) |
| Any related AE | 0 | 0 | 0 |  | 1 (3.4) | 0 | 1 (0.9) | 0 | 1 (0.7) |
| **Any Grade ≥3 AE** | 0 | 2 (6.9) | 0 |  | 1 (3.4) | 0 | 3 (2.6) | 0 | 3 (2.1) |
| Increased blood pressure | 0 | 1 (3.4) | 0 |  | 1 (3.4) | 0 | 2 (1.7) | 0 | 2 (1.4) |
| Hypertension | 0 | 1 (3.4) | 0 |  | 0 | 0 | 1 (0.9) | 0 | 1 (0.7) |
| Any related Grade ≥3 AE | 0 | 0 | 0 |  | 0 | 0 | 0 | 0 | 0 |
| **Any MAAE** | 7 (35.0) | 15 (51.7) | 7 (36.8) |  | 8 (27.6) | 9 (45.0) | 46 (39.3) | 8 (34.8) | 54 (38.6) |
| Any related MAAE | 0 | 0 | 0 |  | 0 | 0 | 0 | 0 | 0 |
| **Any SAE** | 1 (5.0) | 3 (10.3) | 0 |  | 0 | 0 | 4 (3.4) | 1 (4.3) | 5 (3.6) |
| Femur fracture | 0 | 0 | 0 |  | 0 | 0 | 0 | 1 (4.3) | 1 (0.7) |
| Lower limb fracture | 0 | 1 (3.4) | 0 |  | 0 | 0 | 1 (0.9) | 0 | 1 (0.7) |
| Rib fracture | 1 (5.0) | 0 | 0 |  | 0 | 0 | 1 (0.9) | 0 | 1 (0.7) |
| Skin laceration | 1 (5.0) | 0 | 0 |  | 0 | 0 | 1 (0.9) | 0 | 1 (0.7) |
| Myocardial infarction | 0 | 1 (3.4) | 0 |  | 0 | 0 | 1 (0.9) | 0 | 1 (0.7) |
| Urinary tract infection | 0 | 1 (3.4) | 0 |  | 0 | 0 | 1 (0.9) | 0 | 1 (0.7) |
| Balance disorder | 1 (5.0) | 0 | 0 |  | 0 | 0 | 1 (0.9) | 0 | 1 (0.7) |
| Nephrolithiasis | 0 | 1 (3.4) | 0 |  | 0 | 0 | 1 (0.9) | 0 | 1 (0.7) |
| Any related SAE | 0 | 0 | 0 |  | 0 | 0 | 0 | 0 | 0 |
| **Any AESI** | 0 | 0 | 0 |  | 0 | 0 | 0 | 0 | 0 |
| **Any AE leading to trial discontinuation** | 0 | 0 | 0 |  | 0 | 0 | 0 | 0 | 0 |
| **Any deaths** | 0 | 0 | 0 |  | 0 | 0 | 0 | 0 | 0 |

**Table S2. RSV- and hMPV-specific nAb GMTs and GMFRs by baseline nAb titer tertile up to Day 365**

nAb titers against **(A)** RSV A, **(B)** RSV B, **(C)** hMPV A, and **(D)** hMPV B were assessed prior to vaccination on Day 0 (baseline), and post-vaccination on Days 7, 28, 180, and 365 in the per-protocol set (N=122); data for n = 7 participants were excluded at specific timepoints, as summarized in the appendix (p9). GMFRs at each timepoint were calculated from baseline. IVX-A12 dose levels were defined as follows: low = 75 µg RSV/75 µg hMPV; medium = 75 µg RSV/150 µg hMPV; high = 75 µg RSV/225 µg hMPV.

CI, confidence intervals; GMFR, geometric mean fold-rise; GMT, geometric mean titer; hMPV, human metapneumovirus; IU, international units; nAb, neutralizing antibody; RSV, respiratory syncytial virus.

**(A) RSV A, IU/mL**

|  |  | **Unadjuvanted** | | |  | **+ MF59^®^** | |  |  |  |
| --- | --- | --- | --- | --- | --- | --- | --- | --- | --- | --- |
| **Trial visit** | **Summary statistics** | **Low** | **Medium** | **High** |  | **Low** | **Medium** | **IVX-A12 total** | **Placebo** | **Total** |
| **First tertile (baseline titer range: 219–1988)** | | | | | | | | | | |
| Baseline | n | 6 | 6 | 5 |  | 9 | 5 | 31 | 10 | 41 |
|  | GMT (95% CI) | 1096.2 (715.8, 1678.8) | 1235.7 (851.8, 1792.7) | 731.9 (274.2, 1954.2) |  | 1036.6 (654.0, 1642.8) | 946.9 (429.8, 2086.3) | 1010.1 (823.7, 1238.6) | 883.0 (579.8, 1344.8) | 977.5 (819.2, 1166.3) |
| Day 7 | n | 6 | 6 | 4 |  | 9 | 4 | 29 | 10 | 39 |
|  | GMT (95% CI) | 2834.8 (1295.0, 6205.6) | 4197.6 (817.1, 21563.5) | 7973.6 (379.3, 167603.4) |  | 3603.8 (1403.0, 9256.8) | 4969.5 (1104.4, 22362.4) | 4127.8 (2568.6, 6633.5) | 1333.8 (728.3, 2442.7) | 3089.7 (2059.6, 4635.0) |
|  | GMFR (95% CI) | 2.59 (1.08, 6.20) | 3.40 (0.71, 16.28) | 12.06 (0.22, 659.48) |  | 3.48 (1.44, 8.38) | 5.59 (2.03, 15.39) | 4.13 (2.46, 6.93) | 1.51 (1.09, 2.10) | 3.19 (2.11, 4.82) |
| Day 28 | n | 6 | 5 | 5 |  | 9 | 5 | 30 | 10 | 40 |
|  | GMT (95% CI) | 4177.8 (2172.8, 8033.0) | 3083.2 (1089.6, 8724.0) | 7883.4 (3291.0, 18884.1) |  | 5978.1 (2494.1, 14328.5) | 6834.5 (1443.8, 32351.9) | 5336.1 (3737.0, 7619.6) | 1500.4 (549.2, 4099.1) | 3885.7 (2646.6, 5704.9) |
|  | GMFR (95% CI) | 3.81 (1.50, 9.70) | 2.38 (0.72, 7.88) | 10.77 (5.11, 22.70) |  | 5.77 (2.91, 11.44) | 7.22 (2.90, 17.99) | 5.28 (3.74, 7.44) | 1.70 (0.79, 3.67) | 3.98 (2.82, 5.60) |
| Day 180 | n | 5 | 5 | 5 |  | 9 | 3 | 27 | 9 | 36 |
|  | GMT (95% CI) | 1751.8 (1012.6, 3030.7) | 2520.5 (218.5, 29,075.9) | 1172.5 (243.0, 5658.0) |  | 2571.2 (1008.2, 6557.7) | 585.1 (23.4, 14,659.7) | 1750.2 (1044.0, 2934.1) | 997.7 (385.2, 2583.8) | 1520.8 (981.0, 2357.4) |
|  | GMFR (95% CI) | 1.61 (0.61, 4.24) | 1.95 (0.21, 17.68) | 1.60 (0.72, 3.59) |  | 2.48 (1.11, 5.52) | 0.84 (0.08, 8.61) | 1.79 (1.17, 2.74) | 1.23 (0.64, 2.36) | 1.63 (1.15, 2.31) |
| Day 365 | n | 4 | 5 | 5 |  | 8 | 4 | 26 | 9 | 35 |
|  | GMT (95% CI) | 2130.3 (1751.4, 2591.2) | 2901.2 (477.6, 17,621.8) | 1308.5 (498.8, 3432.9) |  | 2552.3 (1329.3, 4900.4) | 1272.7 (200.3, 8084.9) | 2010.3 (1374.9, 2939.3) | 1437.5 (529.6, 3902.1) | 1844.2 (1292.7, 2630.9) |
|  | GMFR (95% CI) | 2.21 (1.17, 4.17) | 2.48 (0.46, 13.47) | 1.79 (1.02, 3.14) |  | 2.62 (1.50, 4.56) | 1.59 (0.47, 5.38) | 2.17 (1.60, 2.96) | 1.77 (0.68, 4.63) | 2.06 (1.52, 2.80) |
| **Second tertile (baseline titer range: 2042–6445)** | | | | | | | | | | |
| Baseline | n | 3 | 7 | 6 |  | 6 | 13 | 35 | 6 | 41 |
|  | GMT (95% CI) | 4042.3 (1483.3, 11,015.9) | 3688.9 (2543.1, 5350.8) | 4051.4 (3281.4, 5002.0) |  | 3564.4 (2549.4, 4983.5) | 3876.5 (3143.5, 4780.6) | 3825.8 (3425.4, 4273.1) | 3879.0 (2579.9, 5832.3) | 3833.6 (3457.6, 4250.4) |
| Day 7 | n | 3 | 7 | 5 |  | 6 | 13 | 34 | 5 | 39 |
|  | GMT (95% CI) | 3814.9 (1355.2, 10,739.3) | 11424.1 (3491.5, 37,378.8) | 8411.4 (999.6, 70,781.5) |  | 5327.2 (2528.6, 11,223.2) | 9407.3 (4684.4, 18,891.8) | 8044.8 (5368.4, 12,055.5) | 2929.4 (1037.8, 8269.3) | 7067.5 (4844.3, 10,311.2) |
|  | GMFR (95% CI) | 0.94 (0.65, 1.37) | 3.10 (0.86, 11.10) | 2.13 (0.23, 19.78) |  | 1.49 (0.63, 3.56) | 2.43 (1.22, 4.82) | 2.11 (1.39, 3.21) | 0.78 (0.28, 2.19) | 1.86 (1.26, 2.74) |
| Day 28 | n | 3 | 7 | 6 |  | 6 | 13 | 35 | 6 | 41 |
|  | GMT (95% CI) | 4765.1 (1013.1, 22,411.4) | 10726.5 (2851.6, 40,348.8) | 24783.2 (5951.7, 10,3198.5) |  | 13413.7 (3628.5, 49,586.6) | 13290.1 (6238.5, 28,312.5) | 12996.2 (8406.9, 20,090.8) | 3166.3 (1123.1, 8926.6) | 10569.9 (6966.8, 16,036.3) |
|  | GMFR (95% CI) | 1.18 (0.59, 2.37) | 2.91 (0.91, 9.32) | 6.12 (1.42, 26.38) |  | 3.76 (1.04, 13.61) | 3.43 (1.72, 6.84) | 3.40 (2.26, 5.11) | 0.82 (0.29, 2.30) | 2.76 (1.85, 4.10) |
| Day 180 | n | 3 | 6 | 6 |  | 6 | 12 | 33 | 5 | 38 |
|  | GMT (95% CI) | 3091.5 (395.0, 24,194.5) | 6467.4 (1528.0, 27,374.3) | 13799.4 (4018.4, 47,387.6) |  | 4834.6 (1289.6, 18,124.7) | 6495.4 (2824.1, 14,939.1) | 6593.8 (4230.3, 10,277.8) | 1469.0 (303.8, 7103.1) | 5411.7 (3484.5, 8404.7) |
|  | GMFR (95% CI) | 0.76 (0.26, 2.22) | 1.77 (0.33, 9.44) | 3.41 (1.01, 11.54) |  | 1.36 (0.33, 5.56) | 1.74 (0.82, 3.67) | 1.75 (1.13, 2.71) | 0.39 (0.10, 1.53) | 1.44 (0.93, 2.21) |
| Day 365 | n | 3 | 6 | 6 |  | 6 | 12 | 33 | 5 | 38 |
|  | GMT (95% CI) | 3380.4 (1506.4, 7585.8) | 4772.4 (1655.1, 13,761.1) | 9328.3 (3949.8, 22,031.1) |  | 3757.7 (1407.4, 10,032.7) | 6899.2 (3295.8, 14,442.3) | 5719.7 (4015.5, 8147.1 | 1728.0 (648.6, 4603.7) | 4886.2 (3463.5, 6893.4) |
|  | GMFR (95% CI) | 0.84 (0.14, 5.05) | 1.31 (0.43, 4.00) | 2.30 (0.95, 5.60) |  | 1.05 (0.35, 3.15) | 1.85 (0.93, 3.64) | 1.52 (1.06, 2.16) | 0.46 (0.14, 1.51) | 1.30 (0.91, 1.84) |
| **Third tertile (baseline titer range: 6654–141,830)** | | | | | | | | | | |
| Baseline | n | 11 | 7 | 8 |  | 7 | 2 | 35 | 5 | 40 |
|  | GMT (95% CI) | 26,536.1 (13,962.6, 50,432.1) | 20,357.3 (10,203.0, 40,617.5) | 9978.9 (7042.1, 14,140.5) |  | 29,768.0 (12,840.2, 69,012.8) | 7462.2 (4095.3, 13,596.9) | 19,152.5 (14,150.5, 25,922.6) | 9419.5 (6310.0, 14,061.4) | 17,526.7 (13,301.3, 23,094.5) |
| Day 7 | n | 11 | 7 | 8 |  | 7 | 2 | 35 | 5 | 40 |
|  | GMT (95% CI) | 33,112.6 (19,363.9, 56,623.0) | 28,588.8 (10,894.8, 75,019.2) | 10,761.2 (4412.4, 26,244.7) |  | 28,330.4 (14,259.7, 56,285.1) | 7544.4 (4036.5, 14,100.7) | 22,151.6 (15,765.6, 31,124.4) | 12,767.6 (5135.4, 31,742.7) | 20,677.3 (15,155.3, 28,211.2) |
|  | GMFR (95% CI) | 1.25 (1.00, 1.56) | 1.40 (0.98, 2.01) | 1.08 (0.40, 2.88) |  | 0.95 (0.64, 1.41) | 1.01 (0.99, 1.04) | 1.16 (0.93, 1.43) | 1.36 (0.53, 3.45) | 1.18 (0.96, 1.45) |
| Day 28 | n | 9 | 6 | 8 |  | 7 | 2 | 32 | 4 | 36 |
|  | GMT (95% CI) | 35,559.4 (16,588.6, 76,225.1) | 29,034.9 (9527.2, 88,486.5) | 18,230.1 (8708.7, 38,161.7) |  | 16,639.6 (4193.2, 66,029.9) | 9669.6 (3686.8, 25,361.2) | 22,615.9 (15,252.4, 33,534.4) | 7637.7 (4132.3, 14,117.0) | 20,046.1 (13,859.5, 28,994.4) |
|  | GMFR (95% CI) | 1.11 (0.74, 1.68) | 1.26 (0.61, 2.58) | 1.83 (0.66, 5.03) |  | 0.56 (0.22, 1.39) | 1.30 (0.90, 1.87) | 1.12 (0.80, 1.56) | 0.92 (0.43, 1.95) | 1.10 (0.81, 1.47) |
| Day 180 | n | 10 | 7 | 8 |  | 7 | 2 | 34 | 5 | 39 |
|  | GMT (95% CI) | 23,270.4 (12,021.2, 45,046.3) | 20,477.5 (5825.4, 71,982.7) | 8698.0 (3957.7, 19,116.0) |  | 29,016.4 (13,028.8, 64,622.1) | 1573.5 (6.7, 369,469.5) | 16,059.2 (10,548.9, 24,447.7) | 5935.5 (3753.3, 9386.5) | 14,135.3 (9648.4, 20,708.7) |
|  | GMFR (95% CI) | 0.78 (0.56, 1.08) | 1.01 (0.53, 1.92) | 0.87 (0.32, 2.39) |  | 0.97 (0.24, 3.92) | 0.21 (0.00, 27.17) | 0.82 (0.58, 1.16) | 0.63 (0.35, 1.14) | 0.79 (0.58, 1.08) |
| Day 365 | n | 9 | 7 | 6 |  | 7 | 2 | 31 | 4 | 35 |
|  | GMT (95% CI) | 21,257.6 (11,487.7, 39,336.3) | 15,128.9 (5377.5, 42,563.0) | 12,396.2 (4662.3, 32,958.8) |  | 17,765.4 (7602.3, 41,515.0) | 2325.8 (667.0, 8110.2) | 14,764.9 (10,200.0, 21,372.7) | 9492.6 (970.4, 92,858.2) | 14,038.0 (9793.3, 20,122.5) |
|  | GMFR (95% CI) | 0.78 (0.49, 1.23) | 0.74 (0.36, 1.55) | 1.32 (0.45, 3.83) |  | 0.60 (0.27, 1.32) | 0.31 (0.16, 0.60) | 0.76 (0.56, 1.02) | 0.92 (0.08, 10.21) | 0.77 (0.57, 1.05) |

**(B) RSV B, IU/mL**

|  |  | **Unadjuvanted** | | |  | **+ MF59^®^** | |  |  |  |
| --- | --- | --- | --- | --- | --- | --- | --- | --- | --- | --- |
| **Trial visit** | **Summary statistics** | **Low** | **Medium** | **High** |  | **Low** | **Medium** | **IVX-A12 total** | **Placebo** | **Total** |
| **First tertile (baseline titer range: 98–1624)** | | | | | | | | | | |
| Baseline | n | 5 | 8 | 7 |  | 9 | 6 | 35 | 8 | 43 |
|  | GMT (95% CI) | 724.8 (372.1, 1411.8) | 812.4 (549.4, 1201.2) | 663.4 (285.1, 1543.5) |  | 890.2 (545.1, 1453.7) | 1035.3 (716.5, 1495.9) | 819.1  (666.0, 1007.4) | 895.2 (564.5, 1419.8) | 832.8 (694.9, 997.9) |
| Day 7 | n | 5 | 8 | 6 |  | 9 | 6 | 34 | 8 | 42 |
|  | GMT (95% CI) | 1779.6 (768.9, 4118.7) | 2644.7 (1032.6, 6773.3) | 2457.4 (1265.5, 4771.8) |  | 2960.5 (1332.4, 6577.6) | 2174.2 (650.4, 7268.4) | 2451.3 (1768.5, 3397.7) | 1045.3 (686.4, 1591.9) | 2084.0 (1560.2, 2783.5) |
|  | GMFR (95% CI) | 2.46 (1.10, 5.49) | 3.26 (1.37, 7.73) | 4.03 (1.38, 11.79) |  | 3.33 (1.52, 7.27) | 2.10 (0.62, 7.07) | 3.02 (2.15, 4.23) | 1.17 (0.83, 1.64) | 2.52 (1.87, 3.40) |
| Day 28 | n | 5 | 7 | 7 |  | 9 | 6 | 34 | 8 | 42 |
|  | GMT (95% CI) | 3317.9 (2244.8, 4903.9) | 2618.4 (1538.0, 4457.9) | 4805.3 (2066.4, 11,174.5) |  | 4452.1 (1780.1, 11,135.3) | 3063.0 (1078.9, 8695.3) | 3634.9 (2673.4, 4942.2) | 813.8 (474.4, 1396.1) | 2733.3 (1986.5, 3760.9) |
|  | GMFR (95% CI) | 4.58 (2.09, 10.02) | 3.39 (2.10, 5.46) | 7.24 (3.29, 15.94) |  | 5.00 (2.18, 11.50) | 2.96 (1.00, 8.73) | 4.48 (3.30, 6.09) | 0.91 (0.53, 1.56) | 3.31 (2.39, 4.58) |
| Day 180 | n | 4 | 7 | 7 |  | 9 | 5 | 32 | 8 | 40 |
|  | GMT (95% CI) | 1172.6 (471.9, 2913.9) | 1320.0 (963.7, 1808.0) | 823.8 (445.0, 1525.1) |  | 1202.4 (669.6, 2159.0) | 1069.4 (468.0, 2443.8) | 1105.8 (886.4, 1379.4) | 622.8 (320.9, 1208.8) | 985.8 (792.5, 1226.3) |
|  | GMFR (95% CI) | 1.98 (0.51, 7.72) | 1.71 (1.15, 2.53) | 1.24 (0.88, 1.75) |  | 1.35 (0.91, 2.01) | 1.03 (0.55, 1.94) | 1.40 (1.16, 1.70) | 0.70 (0.39, 1.23) | 1.22 (1.00, 1.49) |
| Day 365 | n | 3 | 7 | 7 |  | 8 | 5 | 30 | 8 | 38 |
|  | GMT (95% CI) | 924.3 (373.4, 2287.7) | 863.3 (391.6, 1903.1) | 810.6 (601.0, 1093.4) |  | 1000.9 (558.4, 1794.1) | 1098.9 (262.9, 4593.8) | 927.6  (713.3, 1206.2) | 559.7 (295.0, 1062.0) | 834.0 (654.7, 1062.4) |
|  | GMFR (95% CI) | 1.66 (0.58, 4.78) | 0.96 (0.49, 1.88) | 1.22 (0.58, 2.57) |  | 1.19 (0.66, 2.16) | 1.06 (0.17, 6.63) | 1.16 (0.85, 1.58) | 0.63 (0.29, 1.33) | 1.02 (0.76, 1.35) |
| **Second tertile (baseline titer range: 1632–6196)** | | | | | | | | | | |
| Baseline | n | 5 | 4 | 7 |  | 7 | 8 | 31 | 8 | 39 |
|  | GMT (95% CI) | 2941.9 (1764.2, 4905.7) | 2734.3 (1750.9, 4269.9) | 3159.7 (1993.6, 5007.9) |  | 2701.6 (1878.4, 3885.5) | 2435.2 (1729.9, 3428.3) | 2766.9 (2390.2, 3203.0) | 2758.2 (2052.8, 3706.2) | 2765.1 (2440.2, 3133.3) |
| Day 7 | n | 5 | 4 | 6 |  | 7 | 7 | 29 | 7 | 36 |
|  | GMT (95% CI) | 3110.8 (1966.1, 4922.2) | 7140.4 (1671.4, 30504.7) | 3822.3 (1839.1, 7943.8) |  | 4082.8 (2738.2, 6087.7) | 5233.6 (3138.9, 8726.4) | 4407.5 (3498.1, 5553.3) | 2552.6 (1597.5, 4078.6) | 3963.4 (3210.8, 4892.4) |
|  | GMFR (95% CI) | 1.06 (0.68, 1.65) | 2.61 (0.69, 9.94) | 1.30 (0.67, 2.51) |  | 1.51 (1.07, 2.13) | 2.03 (1.46, 2.83) | 1.59 (1.29, 1.97) | 0.94 (0.76, 1.17) | 1.44 (1.20, 1.73) |
| Day 28 | n | 4 | 4 | 7 |  | 7 | 8 | 30 | 8 | 38 |
|  | GMT (95% CI) | 3154.8 (1031.7, 9646.4) | 12,763.4 (1728.4, 94249.4) | 5262.2 (1659.9, 16,682.1) |  | 7988.3 (2906.1, 21,958.6) | 10,090.5 (3372.1, 30,194.6) | 7253.5 (4666.4, 11,274.9) | 3008.7 (1350.9, 6701.0) | 6026.8 (4085.3, 8891.2) |
|  | GMFR (95% CI) | 1.14 (0.66, 1.97) | 4.67 (0.98, 22.30) | 1.67 (0.61, 4.58) |  | 2.96 (1.07, 8.16) | 4.14 (1.66, 10.33) | 2.65 (1.78, 3.95) | 1.09 (0.63, 1.89) | 2.20 (1.55, 3.11) |
| Day 180 | n | 4 | 3 | 7 |  | 7 | 6 | 27 | 6 | 33 |
|  | GMT (95% CI) | 1971.4 (846.6, 4590.6) | 2616.4 (303.6, 22,548.7) | 2090.9 (995.8, 4390.3) |  | 2602.8 (1610.0, 4207.6) | 2777.2 (1554.6, 4961.3) | 2395.6 (1878.1, 3055.7) | 1059.3 (573.5, 1956.7) | 2065.3 (1622.5, 2628.9) |
|  | GMFR (95% CI) | 0.71 (0.55, 0.93) | 1.07 (0.20, 5.68) | 0.66 (0.34, 1.30) |  | 0.96 (0.48, 1.94) | 1.03 (0.74, 1.42) | 0.86 (0.68, 1.08) | 0.38 (0.21, 0.71) | 0.74 (0.59, 0.94) |
| Day 365 | n | 4 | 3 | 6 |  | 7 | 7 | 27 | 6 | 33 |
|  | GMT (95% CI) | 1381.4 (598.6, 3187.6) | 1231.0 (172.0, 8809.2) | 1315.6 (551.4, 3138.7) |  | 1749.0 (980.9, 3118.6) | 1863.9 (1014.6, 3424.1) | 1550.0 (1196.0, 2008.9) | 1440.0 (669.2, 3098.5) | 1529.4 (1211.1, 1931.5) |
|  | GMFR (95% CI) | 0.50 (0.28, 0.89) | 0.50 (0.11, 2.34) | 0.38 (0.18, 0.80) |  | 0.65 (0.34, 1.25) | 0.74 (0.49, 1.11) | 0.56 (0.44, 0.71) | 0.52 (0.20, 1.38) | 0.55 (0.44, 0.69) |
| **Third tertile (baseline titer range: 6263–83,294)** | | | | | | | | | | |
| Baseline | n | 10 | 8 | 5 |  | 6 | 6 | 35 | 5 | 40 |
|  | GMT (95% CI) | 18,652.1 (10,506.4, 33,113.1) | 18,285.7 (8706.8, 38,402.7) | 16,143.2 (6944.5, 37,526.2) |  | 12,421.3 (6594.9, 23,395.4) | 12,814.2 (6425.5, 25,555.1) | 15,906.7 (12,372.1, 20,451.0) | 15,826.5 (5102.5, 49,089.3) | 15,896.6 (12,535.2, 20,159.4) |
| Day 7 | n | 10 | 8 | 5 |  | 6 | 6 | 35 | 5 | 40 |
|  | GMT (95% CI) | 17,965.7 (9073.2, 35,573.5) | 18,238.4 (8975.8, 37,059.5) | 9907.3 (5229.3, 18,769.9) |  | 12,348.2 (7121.2, 21,412.0) | 19,133.8 (9181.3, 39,874.8) | 15,695.9 (12,050.5, 20,444.1) | 14,138.5 (4666.5, 42,836.3) | 15,492.2 (12,095.2, 19,843.3) |
|  | GMFR (95% CI) | 0.96 (0.71, 1.31) | 1.00 (0.83, 1.20) | 0.61 (0.31, 1.22) |  | 0.99 (0.83, 1.19) | 1.49 (0.83, 2.70) | 0.99 (0.84, 1.15) | 0.89 (0.58, 1.38) | 0.97 (0.85, 1.12) |
| Day 28 | n | 9 | 7 | 5 |  | 6 | 6 | 33 | 4 | 37 |
|  | GMT (95% CI) | 16,098.6 (8867.5, 29,226.3) | 19,961.9 (9203.2, 43,298.1) | 25,526.4 (8930.6, 72,962.0) |  | 9977.6 (4258.9, 23,374.9) | 17,025.3 (7251.2, 39,974.3) | 16,733.3 (12,537.5, 22,333.1) | 16,767.9 (2700.5, 104,114.3) | 16,737.0 (12,665.6, 22,117.2) |
|  | GMFR (95% CI) | 0.88 (0.63, 1.24) | 0.94 (0.81, 1.10) | 1.58 (0.61, 4.13) |  | 0.80 (0.55, 1.18) | 1.33 (0.81, 2.18) | 1.04 (0.87, 1.23) | 1.02 (0.65, 1.62) | 1.03 (0.88, 1.21) |
| Day 180 | n | 10 | 8 | 5 |  | 6 | 6 | 35 | 5 | 40 |
|  | GMT (95% CI) | 9151.6 (4762.1, 17,587.1) | 9438.1 (4115.3, 21,645.2) | 5182.4 (2172.3, 12,363.8) |  | 4092.1 (1828.8, 9156.4) | 5122.4 (2113.6, 12,414.6) | 6701.2 (4934.0, 9101.2) | 13,079.4 (1863.1, 91,820.1) | 7285.4 (5295.4, 10,023.3) |
|  | GMFR (95% CI) | 0.49 (0.32, 0.76) | 0.52 (0.32, 0.83) | 0.32 (0.19, 0.55) |  | 0.33 (0.14, 0.80) | 0.40 (0.23, 0.70) | 0.42 (0.34, 0.52) | 0.83 (0.23, 2.94) | 0.46 (0.37, 0.57) |
| Day 365 | n | 9 | 8 | 4 |  | 6 | 6 | 33 | 4 | 37 |
|  | GMT (95% CI) | 4872.2 (2782.2, 8532.5) | 8290.9 (4280.7, 16,057.8) | 8208.4 (1514.7, 44482.1) |  | 3978.8 (1532.1, 10,332.9) | 3507.0 (891.7, 13,792.4) | 5360.4 (3833.6, 7495.3) | 7443.7 (975.4, 56,806.2) | 5554.1 (4019.2, 7675.2) |
|  | GMFR (95% CI) | 0.28 (0.16, 0.51) | 0.45 (0.28, 0.74) | 0.49 (0.26, 0.93) |  | 0.32 (0.12, 0.88) | 0.27 (0.12, 0.63) | 0.35 (0.27, 0.45) | 0.70 (0.10, 4.67) | 0.37 (0.29, 0.49) |

**(C) hMPV A, assay units/mL**

|  |  | **Unadjuvanted** | | |  | **+ MF59^®^** | |  |  |  |
| --- | --- | --- | --- | --- | --- | --- | --- | --- | --- | --- |
| **Trial visit** | **Summary statistics** | **Low** | **Medium** | **High** |  | **Low** | **Medium** | **IVX-A12 total** | **Placebo** | **Total** |
| **First tertile (baseline titer range: 132–544)** | | | | | | | | | | |
| Baseline | n | 3 | 3 | 10 |  | 8 | 9 | 33 | 8 | 41 |
|  | GMT (95% CI) | 335.8 (162.2, 695.3) | 275.5 (129.3, 586.9) | 278.8 (208.0, 373.5) |  | 344.4 (257.2, 461.1) | 295.3 (218.5, 399.0) | 302.8  (266.3, 344.4) | 319.3 (259.6, 392.7) | 306.0 (274.7, 340.8) |
| Day 7 | n | 3 | 3 | 8 |  | 8 | 8 | 30 | 8 | 38 |
|  | GMT (95% CI) | 1165.3 (167.3, 8117.5) | 731.2  (62.6, 8537.8) | 3988.9 (1387.5, 11,467.4) |  | 1417.7 (399.5, 5031.4) | 1035.1 (580.0, 1847.3) | 1576.5 (994.1, 2500.1) | 466.0 (254.3, 854.1) | 1219.8 (810.3, 1836.0) |
|  | GMFR (95% CI) | 3.47 (0.82, 14.62) | 2.65 (0.32, 22.28) | 12.24 (3.94, 38.01) |  | 4.12 (1.07, 15.91) | 3.39 (1.98, 5.80) | 4.92 (3.08, 7.86) | 1.46 (0.86, 2.47) | 3.81 (2.53, 5.74) |
| Day 28 | n | 3 | 3 | 10 |  | 8 | 9 | 33 | 8 | 41 |
|  | GMT (95% CI) | 1288.5 (264.6, 6273.1) | 2102.3 (150.3, 29,405.1) | 2578.6 (1361.3, 4884.2) |  | 3262.5 (962.9, 11,054.3) | 1324.3 (567.1, 3092.2) | 2097.8 (1414.7, 3110.7) | 389.6 (198.9, 763.3) | 1510.4 (1018.7, 2239.5) |
|  | GMFR (95% CI) | 3.84 (1.37, 10.71) | 7.63 (0.58, 100.97) | 9.25 (4.45, 19.21) |  | 9.47 (2.31, 38.92) | 4.48 (2.09, 9.62) | 6.93 (4.57, 10.51) | 1.22 (0.65, 2.31) | 4.94 (3.27, 7.44) |
| Day 180 | n | 3 | 3 | 10 |  | 8 | 6 | 30 | 8 | 38 |
|  | GMT (95% CI) | 597.2  (97.2, 3668.6) | 670.9 (280.8, 1603.0) | 1135.4 (679.5, 1897.2) |  | 843.3 (432.2, 1645.2) | 778.9 (458.3, 1324.0) | 865.4  (673.2, 1112.5) | 391.3 (223.9, 683.7) | 732.2 (574.3, 933.6) |
|  | GMFR (95% CI) | 1.78 (0.46, 6.86) | 2.43 (1.46, 4.06) | 4.07 (2.73, 6.07) |  | 2.45 (1.06, 5.68) | 2.18 (1.37, 3.49) | 2.74 (2.11, 3.56) | 1.23 (0.66, 2.27) | 2.32 (1.80, 2.98) |
| Day 365 | n | 3 | 3 | 9 |  | 8 | 7 | 30 | 8 | 38 |
|  | GMT (95% CI) | 258.6  (66.7, 1002.8) | 276.5 (195.2, 391.5) | 529.7 (303.9, 923.2) |  | 503.2 (331.5, 764.0) | 371.6 (249.0, 554.6) | 419.6  (337.8, 521.1) | 294.7 (176.1, 493.2) | 389.5 (320.1, 474.0) |
|  | GMFR (95% CI) | 0.77 (0.29, 2.04) | 1.00 (0.45, 2.26) | 1.97 (1.16, 3.34) |  | 1.46 (0.84, 2.54) | 1.11 (0.91, 1.36) | 1.35 (1.08, 1.69) | 0.92 (0.53, 1.62) | 1.25 (1.02, 1.54) |
| **Second tertile (baseline titer range: 547–1356)** | | | | | | | | | | |
| Baseline | n | 8 | 11 | 7 |  | 5 | 8 | 39 | 3 | 42 |
|  | GMT (95% CI) | 939.1 (725.7, 1215.3) | 757.6 (622.6, 921.9) | 748.1 (535.8, 1044.5) |  | 844.8 (680.2, 1049.2) | 853.9 (668.1, 1091.2) | 821.0  (746.0, 903.5) | 816.3 (452.8, 1471.5) | 820.6 (749.9, 898.0) |
| Day 7 | n | 8 | 11 | 7 |  | 5 | 8 | 39 | 3 | 42 |
|  | GMT (95% CI) | 817.6 (609.5, 1096.8) | 1629.1 (636.3, 4171.0) | 2092.6 (682.7, 6414.1) |  | 1045.6 (327.4, 3338.9) | 2216.1 (1009.8, 4863.4) | 1488.6 (1047.7, 2114.9) | 1064.8 (136.4, 8312.8) | 1453.4 (1043.7, 2023.8) |
|  | GMFR (95% CI) | 0.87 (0.59, 1.28) | 2.15 (0.86, 5.40) | 2.80 (1.03, 7.59) |  | 1.24 (0.42, 3.65) | 2.60 (1.27, 5.31) | 1.81 (1.28, 2.56) | 1.30 (0.11, 14.95) | 1.77 (1.28, 2.46) |
| Day 28 | n | 7 | 10 | 7 |  | 5 | 8 | 37 | 2 | 39 |
|  | GMT (95% CI) | 1505.5 (877.9, 2581.9) | 3245.2 (1451.5, 7255.4) | 1922.7 (1208.9, 3057.9) |  | 2131.1 (1044.5, 4347.9) | 2209.2 (991.3, 4923.2) | 2209.7 (1670.5, 2922.9) | 649.6 (13.9, 30,330.4) | 2075.2 (1568.3, 2746.0) |
|  | GMFR (95% CI) | 1.51 (0.74, 3.06) | 4.25 (1.91, 9.48) | 2.57 (2.03, 3.25) |  | 2.52 (1.49, 4.28) | 2.59 (1.21, 5.52) | 2.66 (2.00, 3.53) | 0.86 (0.00, 446.40) | 2.51 (1.89, 3.33) |
| Day 180 | n | 7 | 10 | 7 |  | 5 | 8 | 37 | 3 | 40 |
|  | GMT (95% CI) | 558.5 (369.5, 844.4) | 1154.9 (660.2, 2020.4) | 1100.6 (587.9, 2060.4) |  | 871.4 (374.4, 2028.1) | 1450.7 (725.9, 2899.2) | 1008.8 (787.0, 1293.0) | 908.7 (245.6, 3362.4) | 1000.9 (793.6, 1262.4) |
|  | GMFR (95% CI) | 0.56 (0.37, 0.84) | 1.51 (0.83, 2.76) | 1.47 (1.06, 2.04) |  | 1.03 (0.51, 2.10) | 1.70 (0.93, 3.09) | 1.21 (0.95, 1.55) | 1.11 (0.36, 3.44) | 1.21 (0.96, 1.51) |
| Day 365 | n | 5 | 10 | 6 |  | 5 | 7 | 33 | 3 | 36 |
|  | GMT (95% CI) | 354.0 (224.5, 558.3) | 725.4 (398.0, 1322.2) | 656.7 (316.2, 1363.7) |  | 483.8 (224.7, 1041.5) | 1290.9 (393.7, 4232.7) | 679.1  (492.3, 936.9) | 518.7 (34.5, 7799.9) | 664.1 (488.2, 903.3) |
|  | GMFR (95% CI) | 0.36 (0.20, 0.62) | 0.93 (0.48, 1.81) | 0.96 (0.56, 1.64) |  | 0.57 (0.29, 1.13) | 1.42 (0.46, 4.42) | 0.82 (0.60, 1.14) | 0.64 (0.05, 8.90) | 0.80 (0.59, 1.10) |
| **Third tertile (baseline titer range: 1361–65,536)** | | | | | | | | | | |
| Baseline | n | 9 | 6 | 2 |  | 9 | 3 | 29 | 10 | 39 |
|  | GMT (95% CI) | 5136.1 (2060.5, 12,802.8) | 3362.4 (1367.1, 8270.0) | 2439.3 (919.4, 6471.6) |  | 2371.2 (1212.2, 4638.2) | 1810.3 (862.3, 3800.6) | 3156.8 (2200.3, 4529.0) | 2502.5 (1421.8, 4404.5) | 2974.3 (2216.9, 3990.4) |
| Day 7 | n | 9 | 6 | 2 |  | 9 | 3 | 29 | 9 | 38 |
|  | GMT (95% CI) | 3874.9 (1547.8, 9701.1) | 2971.7 (730.8, 12,084.7) | 4082.4  (0.0, 1.427E+11) |  | 2362.8 (823.1, 6782.9) | 2203.5 (292.2, 16,617.6) | 2978.2 (1857.9, 4773.9) | 2259.2 (985.7, 5178.2) | 2789.5 (1882.8, 4132.9) |
|  | GMFR (95% CI) | 0.75 (0.43, 1.31) | 0.88 (0.44, 1.78) | 1.67 (0.00, 1.552E+09) |  | 1.00 (0.26, 3.89) | 1.22 (0.18, 8.27) | 0.94 (0.60, 1.48) | 0.84 (0.40, 1.77) | 0.92 (0.63, 1.33) |
| Day 28 | n | 8 | 5 | 2 |  | 9 | 3 | 27 | 10 | 37 |
|  | GMT (95% CI) | 5231.2 (1893.6, 14,451.1) | 4505.2 (651.5, 31,153.3) | 3992.1  (0.0, 8.543E+11) |  | 3109.6 (1213.2, 7970.2) | 2235.9 (285.9, 17,486.2) | 3815.7 (2317.7, 6282.0) | 1852.0 (1149.8, 2983.0) | 3138.5 (2126.2, 4632.8) |
|  | GMFR (95% CI) | 1.01 (0.44, 2.34) | 1.14 (0.41, 3.20) | 1.64 (0.00, 9.292E+09) |  | 1.31 (0.52, 3.28) | 1.24 (0.21, 7.18) | 1.20 (0.79, 1.82) | 0.74 (0.41, 1.34) | 1.05 (0.75, 1.47) |
| Day 180 | n | 8 | 5 | 2 |  | 9 | 3 | 27 | 8 | 35 |
|  | GMT (95% CI) | 1688.9 (623.9, 4571.5) | 2030.6 (488.3, 8443.8) | 2266.9  (0.0, 6.617E+10) |  | 1382.5 (787.3, 2427.7) | 1756.0 (191.9, 16,065.0) | 1678.0 (1134.6, 2481.7) | 866.0 (380.5, 1970.8) | 1442.5 (1018.4, 2043.2) |
|  | GMFR (95% CI) | 0.28 (0.13, 0.58) | 0.67 (0.44, 1.01) | 0.93 (0.00, 7.197E+07) |  | 0.58 (0.32, 1.06) | 0.97 (0.12, 8.14) | 0.53 (0.37, 0.76) | 0.32 (0.11, 0.90) | 0.47 (0.33, 0.66) |
| Day 365 | n | 8 | 5 | 2 |  | 8 | 3 | 26 | 7 | 33 |
|  | GMT (95% CI) | 1043.1 (591.6, 1839.4) | 907.2 (120.6, 6825.3) | 1184.1  (0.0, 1.049E+09) |  | 842.5 (478.2, 1484.5) | 1200.8 (456.3, 3159.7) | 975.9  (679.6, 1401.5) | 1190.5 (409.4, 3462.0) | 1018.0 (729.4, 1420.7) |
|  | GMFR (95% CI) | 0.17 (0.07, 0.44) | 0.30 (0.11, 0.81) | 0.49 (0.00, 1.141E+06) |  | 0.33 (0.19, 0.58) | 0.66 (0.12, 3.56) | 0.30 (0.20, 0.44) | 0.41 (0.12, 1.43) | 0.32 (0.22, 0.46) |

**(D) hMPV B, assay units/mL**

|  |  | **Unadjuvanted** | | |  | **+ MF59^®^** | |  |  |  |
| --- | --- | --- | --- | --- | --- | --- | --- | --- | --- | --- |
| **Trial visit** | **Summary statistics** | **Low** | **Medium** | **High** |  | **Low** | **Medium** | **IVX-A12 total** | **Placebo** | **Total** |
| **First tertile (baseline titer range: 310–4715)** | | | | | | | | | | |
| Baseline | n | 8 | 3 | 8 |  | 8 | 9 | 36 | 5 | 41 |
|  | GMT (95% CI) | 2468.9 (1572.1, 3877.3) | 1902.0 (1604.9, 2254.2) | 2752.4 (1928.5, 3928.1) |  | 2250.7 (1539.2, 3291.3) | 1398.9 (664.2, 2946.1) | 2103.5 (1690.3, 2617.7) | 2593.7 (984.6, 6832.6) | 2157.9 (1753.9, 2654.9) |
| Day 7 | n | 8 | 3 | 6 |  | 8 | 9 | 34 | 5 | 39 |
|  | GMT (95% CI) | 7189.5 (4639.9, 11,140.1) | 16,254.4 (3595.7, 73,477.7) | 26,652.3 (20,310.2, 34,974.8) |  | 20,622.7 (12,895.9, 32,979.3) | 7432.2 (2422.5, 22,801.6) | 12,585.6 (8890.4, 17,816.6) | 5302.4 (1715.2, 16,391.5) | 11,265.4 (8101.4, 15,665.0) |
|  | GMFR (95% CI) | 2.91 (1.83, 4.64) | 8.55 (2.23, 32.75) | 10.08 (5.10, 19.90) |  | 9.16 (4.70, 17.88) | 5.31 (2.56, 11.02) | 6.12 (4.54, 8.26) | 2.04 (0.85, 4.89) | 5.32 (3.96, 7.14) |
| Day 28 | n | 7 | 2 | 8 |  | 8 | 9 | 34 | 5 | 39 |
|  | GMT (95% CI) | 18,437.5 (9542.7, 35,623.0) | 15,600.3 (1.3, 1.943E+08) | 20,280.5 (12,789.9, 32,158.0) |  | 20,066.1 (12,484.0, 32,253.1) | 8894.9 (3160.6, 25,033.0) | 15,704.4 (11,446.1, 21,547.0) | 3746.3 (1382.9, 10,148.5) | 13,068.5 (9424.3, 18,121.8) |
|  | GMFR (95% CI) | 6.44 (2.77, 14.98) | 7.99 (0.00, 51938.14) | 7.37 (3.84, 14.13) |  | 8.92 (4.30, 18.47) | 6.36 (3.34, 12.12) | 7.24 (5.46, 9.62) | 1.44 (0.78, 2.69) | 5.89 (4.33, 8.01) |
| Day 180 | n | 8 | 2 | 8 |  | 8 | 7 | 33 | 5 | 38 |
|  | GMT (95% CI) | 9696.8 (3742.6, 25,123.7) | 3514.2 (209.7, 58,884.0) | 10,077.3 (4392.3, 23,120.6) |  | 8696.4 (3746.9, 20,184.0) | 4638.0 (1453.1, 14,803.6) | 7665.8 (5241.5, 11,211.5) | 1587.3 (270.2, 9323.8) | 6231.2 (4163.5, 9326.0) |
|  | GMFR (95% CI) | 3.93 (1.61, 9.60) | 1.80 (0.06, 57.80) | 3.66 (1.27, 10.58) |  | 3.86 (1.31, 11.37) | 2.94 (1.33, 6.52) | 3.45 (2.36, 5.05) | 0.61 (0.17, 2.22) | 2.75 (1.85, 4.09) |
| Day 365 | n | 7 | 3 | 8 |  | 7 | 7 | 32 | 4 | 36 |
|  | GMT (95% CI) | 4634.1 (2379.2, 9026.1) | 4955.8 (1162.9, 21,120.0) | 7747.4 (4024.5, 14,914.0) |  | 4202.6 (1782.3, 9909.7) | 3615.4 (1265.8, 10,325.7) | 4916.2 (3591.8, 67,28.9) | 1318.6 (247.2, 7034.5) | 4247.4 (3058.4, 5898.6) |
|  | GMFR (95% CI) | 1.93 (1.12, 3.33) | 2.61 (0.57, 11.86) | 2.81 (1.18, 6.71) |  | 1.88 (0.65, 5.45) | 2.29 (0.90, 5.82) | 2.25 (1.63, 3.12) | 0.58 (0.39, 0.87) | 1.94 (1.40, 2.68) |
| **Second tertile (baseline titer range: 4923–15,850)** | | | | | | | | | | |
| Baseline | n | 7 | 7 | 7 |  | 7 | 6 | 34 | 7 | 41 |
|  | GMT (95% CI) | 9843.5 (6424.7, 15,081.6) | 9222.1 (7128.8, 11,930.0) | 8024.5 (5212.3, 12,353.8) |  | 9781.4 (7141.9, 13,396.4) | 8170.9 (5609.1, 11,902.7) | 8999.4 (7895.6, 10,257.5) | 8728.0 (6836.5, 11,142.8) | 8952.4 (8001.3, 10,016.6) |
| Day 7 | n | 7 | 7 | 7 |  | 7 | 5 | 33 | 6 | 39 |
|  | GMT (95% CI) | 16,287.1 (8631.3, 30,733.4) | 20,833.6 (13,715.1, 31,646.9) | 27,063.9 (21,234.2, 34,494.1) |  | 22,012.0 (12,576.7, 38,526.1) | 23,063.5 (11,920.4, 44,623.1) | 21,475.7 (17,842.8, 25,848.2) | 11,021.0 (3641.6, 33,354.2) | 19,380.9 (15,646.4, 24,006.7) |
|  | GMFR (95% CI) | 1.65 (1.10, 2.50) | 2.26 (1.59, 3.21) | 3.37 (2.11, 5.40) |  | 2.25 (1.41, 3.58) | 2.68 (2.02, 3.55) | 2.36 (1.99, 2.79) | 1.19 (0.45, 3.14) | 2.13 (1.75, 2.58) |
| Day 28 | n | 6 | 6 | 7 |  | 7 | 6 | 32 | 6 | 38 |
|  | GMT (95% CI) | 14,593.2 (8757.2, 24,318.6) | 19,186.7 (8463.7, 43,495.2) | 24,299.2 (15,939.8, 37,042.6) |  | 26,181.6 (18,964.6, 36,144.9) | 19,254.3 (9310.5, 39,818.0) | 20,557.7 (16,757.6, 25,219.6) | 9080.4 (4040.3, 20,407.6) | 18,069.3 (14,526.4, 22,476.4) |
|  | GMFR (95% CI) | 1.61 (0.95, 2.70) | 2.12 (1.16, 3.88) | 3.03 (1.77, 5.19) |  | 2.68 (2.20, 3.25) | 2.36 (1.32, 4.21) | 2.34 (1.94, 2.81) | 1.02 (0.58, 1.78) | 2.05 (1.68, 2.49) |
| Day 180 | n | 6 | 7 | 7 |  | 7 | 5 | 32 | 6 | 38 |
|  | GMT (95% CI) | 15,055.6 (5986.9, 37,860.9) | 9524.7 (2925.6, 31,009.6) | 19,100.0 (10,159.0, 35,910.0) |  | 17,433.2 (8131.8, 37,373.8) | 19,458.0 (7827.7, 48,368.5) | 15,422.0 (11,157.5, 21,316.5) | 11,433.5 (7030.2, 18,594.6) | 14,710.3 (11,137.4, 19,429.3) |
|  | GMFR (95% CI) | 1.66 (0.92, 3.00) | 1.03 (0.37, 2.92) | 2.38 (1.22, 4.66) |  | 1.78 (1.04, 3.06) | 2.26 (1.40, 3.65) | 1.72 (1.31, 2.27) | 1.24 (0.80, 1.91) | 1.64 (1.29, 2.08) |
| Day 365 | n | 5 | 7 | 6 |  | 7 | 6 | 31 | 6 | 37 |
|  | GMT (95% CI) | 5721.1 (1637.1, 19,993.3) | 4060.8 (764.0, 21,584.1) | 8217.2 (3468.9, 19,465.0) |  | 13,082.1 (5385.7, 31,777.0) | 10,341.4 (4121.4, 25,948.7) | 7676.6 (4968.5, 11,860.7) | 4744.3 (2458.2, 9156.7) | 7100.3 (4884.2, 10,322.0) |
|  | GMFR (95% CI) | 0.62 (0.30, 1.26) | 0.44 (0.10, 1.90) | 1.14 (0.41, 3.19) |  | 1.34 (0.63, 2.83) | 1.27 (0.62, 2.58) | 0.88 (0.60, 1.30) | 0.51 (0.27, 0.96) | 0.81 (0.58, 1.13) |
| **Third tertile (baseline titer range: 15,968–32,768)** | | | | | | | | | | |
| Baseline | n | 5 | 10 | 4 |  | 7 | 5 | 31 | 9 | 40 |
|  | GMT (95% CI) | 27,220.8 (19,692.7, 37,626.9) | 27,926.3 (23,915.3, 32,610.2) | 27,692.2 (20,010.6, 38,322.7) |  | 25,066.8 (20,973.1, 29,959.5) | 28,379.8 (19,039.2, 42,303.0) | 27,182.2 (25,027.9, 29,521.9) | 28,156.8 (24,769.9, 32,006.7) | 27,398.5 (25,603.2, 29,319.6) |
| Day 7 | n | 5 | 10 | 4 |  | 7 | 5 | 31 | 9 | 40 |
|  | GMT (95% CI) | 25,908.2 (14,333.1, 46,831.1) | 27,875.3 (22,582.1, 34,409.2) | 30,619.5 (26,455.3, 35,439.3) |  | 21,734.5 (13,882.9, 34,026.6) | 24,614.7 (11,729.1, 51,656.5) | 25,836.8 (22,262.4, 29,985.1) | 27,674.3 (22,473.4, 34,078.9) | 26,239.3 (23,253.8, 29,608.1) |
|  | GMFR (95% CI) | 0.95 (0.71, 1.27) | 1.00 (0.80, 1.24) | 1.11 (0.75, 1.63) |  | 0.87 (0.51, 1.47) | 0.87 (0.62, 1.22) | 0.95 (0.84, 1.08) | 0.98 (0.78, 1.24) | 0.96 (0.86, 1.07) |
| Day 28 | n | 5 | 10 | 4 |  | 7 | 5 | 31 | 9 | 40 |
|  | GMT (95% CI) | 29,748.5 (22,745.5, 38,907.6) | 25,315.2 (16,596.1, 38,615.0) | 32,538.1 (31,817.1, 33,275.4) |  | 25,653.7 (19,168.4, 34,333.1) | 21,103.7 (6318.5, 70,486.0) | 26,140.1 (21,576.3, 31,669.3) | 25,335.5 (20,432.4, 31,415.2) | 25,956.9 (22,290.3, 30,226.5) |
|  | GMFR (95% CI) | 1.09 (0.71, 1.67) | 0.91 (0.63, 1.31) | 1.17 (0.84, 1.65) |  | 1.02 (0.68, 1.55) | 0.74 (0.33, 1.67) | 0.96 (0.81, 1.14) | 0.90 (0.71, 1.14) | 0.95 (0.83, 1.09) |
| Day 180 | n | 4 | 9 | 4 |  | 7 | 5 | 29 | 8 | 37 |
|  | GMT (95% CI) | 27,540.2 (15,839.7, 47,883.7) | 25,623.9 (19,184.7, 34,224.4) | 28,118.2 (19,505.7, 40,533.4) |  | 17,186.0 (10,278.2, 28,736.4) | 23,735.8 (10,558.1, 53,360.8) | 23,492.4 (19,643.9, 28,094.7) | 20,532.5 (10,650.1, 39,584.9) | 22,818.2 (19,038.0, 27,349.0) |
|  | GMFR (95% CI) | 0.90 (0.57, 1.43) | 0.93 (0.76, 1.15) | 1.02 (0.65, 1.59) |  | 0.69 (0.37, 1.26) | 0.84 (0.55, 1.26) | 0.86 (0.73, 1.00) | 0.73 (0.40, 1.33) | 0.83 (0.70, 0.97) |
| Day 365 | n | 4 | 6 | 3 |  | 7 | 5 | 25 | 8 | 33 |
|  | GMT (95% CI) | 21,233.3 (8721.2, 51,696.4) | 14,872.1 (5618.9, 39,363.6) | 22,041.8 (5973.9, 81,326.7) |  | 8258.1 (4940.8, 13,802.8) | 16,564.3 (3930.5, 69,806.0) | 14,303.5 (10,165.7, 20,125.6) | 17,216.6 (9210.1, 32,183.5) | 14,961.0 (11,260.2, 19,878.1) |
|  | GMFR (95% CI) | 0.70 (0.32, 1.53) | 0.54 (0.26, 1.09) | 0.73 (0.29, 1.88) |  | 0.33 (0.18, 0.60) | 0.58 (0.20, 1.69) | 0.52 (0.39, 0.68) | 0.61 (0.32, 1.18) | 0.54 (0.42, 0.69) |

## Table S3. Linear regression for IVX-A12 formulation selection and dose level guidance

Linear regression analyses were performed to assess the effect of adjuvant and dose level on RSV- and hMPV-specific nAb and pre-F IgG titers at Day 28, after adjusting for baseline titer and sex. For each analyte, a series of regression models were fitted to the data. The full model included adjuvant and dose level as categorical variables along with adjuvant by dose level interaction terms. The first reduced model excluded the interaction terms. The second reduced model further excluded the variable for adjuvant. The third reduced model further excluded variables for dose level. The likelihood ratio test was used to determine the significance of the variable(s) being excluded between models. Placebo recipients were excluded from the analysis.
**P* < .05

***P* < .01

hMPV, human metapneumovirus; IgG, immunoglobulin G; nAb, neutralizing antibody; pre-F, prefusion conformation of the fusion protein; RSV, respiratory syncytial virus.

|  | **Likelihood ratio** | |
| --- | --- | --- |
| **Variable** | **Test statistic** | ***P* value** |
| **RSV A nAbs** |  |  |
| Adjuvant x dose level interaction | 0.26 | .610 |
| Adjuvant | 1.01 | .314 |
| Dose level | 3.91 | .048* |
| **RSV B nAbs** |  |  |
| Adjuvant x dose level interaction | 0.29 | .591 |
| Adjuvant | 1.06 | .302 |
| Dose level | 1.54 | .214 |
| **hMPV A nAbs** |  |  |
| Adjuvant x dose level interaction | 2.45 | .118 |
| Adjuvant | 0.01 | .919 |
| Dose level | 0.39 | .534 |
| **hMPV B nAbs** |  |  |
| Adjuvant x dose level interaction | 1.50 | .220 |
| Adjuvant | 0.04 | .833 |
| Dose level | 5.64 | .018* |
| **RSV pre-F IgG** |  |  |
| Adjuvant x dose level interaction | 0.96 | .327 |
| Adjuvant | 0.74 | .389 |
| Dose level | 0.96 | .327 |
| **hMPV pre-F IgG** |  |  |
| Adjuvant x dose level interaction | 0.55 | .458 |
| Adjuvant | 0.10 | .755 |
| Dose level | 10.70 | .001** |

## Table S4. GMRs to assess selectivity for nAb or pre-F IgG responses against RSV and hMPV

GMRs for RSV-specific immune responses were estimated using fold-rise in RSV pre-F IgG titers from baseline divided by fold-rise in RSV A nAb titers from baseline, for each timepoint. GMRs for hMPV-specific immune responses were estimated using fold-rise in hMPV pre-F IgG titers from baseline divided by fold-rise in hMPV A nAb titers from baseline, for each timepoint. These analyses were performed in the per-protocol set (N = 122); data for n = 7 participants were excluded at specific timepoints, as summarized in the appendix (p9). IVX-A12 dose levels were defined as follows: low = 75 µg RSV/75 µg hMPV; medium = 75 µg RSV/150 µg hMPV; high = 75 µg RSV/225 µg hMPV.

CI, confidence interval; GMR, geometric mean ratio; hMPV, human metapneumovirus; IgG, immunoglobulin G; nAb, neutralizing antibody; pre-F, prefusion conformation of the fusion protein; RSV, respiratory syncytial virus.

|  |  | **Unadjuvanted** | | |  | **+ MF59^®^** | |  |  |  |
| --- | --- | --- | --- | --- | --- | --- | --- | --- | --- | --- |
| **Trial visit** | **Summary statistics** | **Low** | **Medium** | **High** |  | **Low** | **Medium** | **IVX-A12 total** | **Placebo** | **Total** |
| **RSV** | | | | | | | | | | |
| Day 7 | n | 20 | 20 | 17 |  | 22 | 19 | 98 | 20 | 118 |
|  | GMR  (95% CI) | 0.79  (0.66, 0.94) | 0.83  (0.56, 1.24) | 0.80  (0.37, 1.72) |  | 1.14  (0.82, 1.59) | 0.90 (0.52, 1.57) | 0.89 (0.74, 1.08) | 0.80 (0.58, 1.09) | 0.87  (0.74, 1.03) |
| Day 28 | n | 18 | 18 | 19 |  | 22 | 20 | 97 | 20 | 117 |
|  | GMR  (95% CI) | 1.00 (0.85, 1.18) | 1.14 (0.68, 1.90) | 0.53  (0.34, 0.83) |  | 1.06 (0.64, 1.78) | 0.91 (0.56, 1.49) | 0.90 (0.74, 1.10) | 0.80 (0.48, 1.32) | 0.88 (0.73, 1.06) |
| Day 180 | n | 18 | 18 | 19 |  | 22 | 17 | 94 | 19 | 113 |
|  | GMR  (95% CI) | 0.85  (0.68, 1.05) | 0.89  (0.48, 1.66) | 0.67  (0.42, 1.06) |  | 0.80  (0.53, 1.19) | 0.89  (0.46, 1.71) | 0.81  (0.66, 0.99) | 1.01  (0.66, 1.56) | 0.84  (0.70, 1.01) |
| Day 365 | n | 16 | 18 | 17 |  | 21 | 18 | 90 | 18 | 108 |
|  | GMR  (95% CI) | 0.60 (0.43, 0.83) | 0.84  (0.53, 1.31) | 0.49  (0.36, 0.68) |  | 0.83  (0.62, 1.12) | 0.57  (0.32, 1.03) | 0.66 (0.55, 0.79) | 0.88 (0.54, 1.44) | 0.69  (0.59, 0.82) |
| **hMPV** | | | | | | | | | | |
| Day 7 | n | 20 | 20 | 17 |  | 22 | 19 | 98 | 20 | 118 |
|  | GMR  (95% CI) | 1.87  (1.28, 2.73) | 1.69 (1.08, 2.65) | 0.50 (0.27, 0.93) |  | 0.94 (0.41, 2.17) | 0.87 (0.61, 1.25) | 1.08 (0.83, 1.39) | 0.88  (0.55, 1.39) | 1.04 (0.83, 1.30) |
| Day 28 | n | 18 | 18 | 19 |  | 22 | 20 | 97 | 20 | 117 |
|  | GMR  (95% CI) | 2.07 (1.34, 3.19) | 1.18 (0.72, 1.94) | 0.91 (0.50, 1.66) |  | 0.84 (0.43, 1.63) | 1.27 (0.81, 1.99) | 1.17 (0.92, 1.48) | 1.21 (0.75, 1.96) | 1.18 (0.96, 1.45) |
| Day 180 | n | 18 | 18 | 19 |  | 22 | 17 | 94 | 19 | 113 |
|  | GMR  (95% CI) | 4.15 (2.40, 7.18) | 1.28 (0.84, 1.95) | 0.83 (0.51, 1.33) |  | 1.09 (0.64, 1.86) | 1.15 (0.76, 1.73) | 1.39 (1.10, 1.75) | 0.98 (0.51, 1.89) | 1.31 (1.05, 1.63) |
| Day 365 | n | 16 | 18 | 17 |  | 21 | 17 | 89 | 18 | 107 |
|  | GMR  (95% CI) | 6.93 (4.52, 10.62) | 2.46 (1.52, 3.98) | 1.11 (0.62, 1.99) |  | 2.05 (1.20, 3.48) | 1.35 (0.89, 2.05) | 2.17 (1.70, 2.77) | 1.46 (0.74, 2.88) | 2.03 (1.62, 2.56) |

# Supplementary Figures

## Figure S1. Study design

Three dose levels of IVX-A12 ± MF59^®^ adjuvant were evaluated using a stepwise dose-escalation approach across three consecutive cohorts. IVX-A12 dose levels were defined as follows: low = 75 µg RSV/75 µg hMPV; medium = 75 µg RSV/150 µg hMPV; high = 75 µg RSV/225 µg hMPV. N numbers represent the number of participants treated in each cohort.

hMPV, human metapneumovirus; R, randomization; RSV, respiratory syncytial virus.


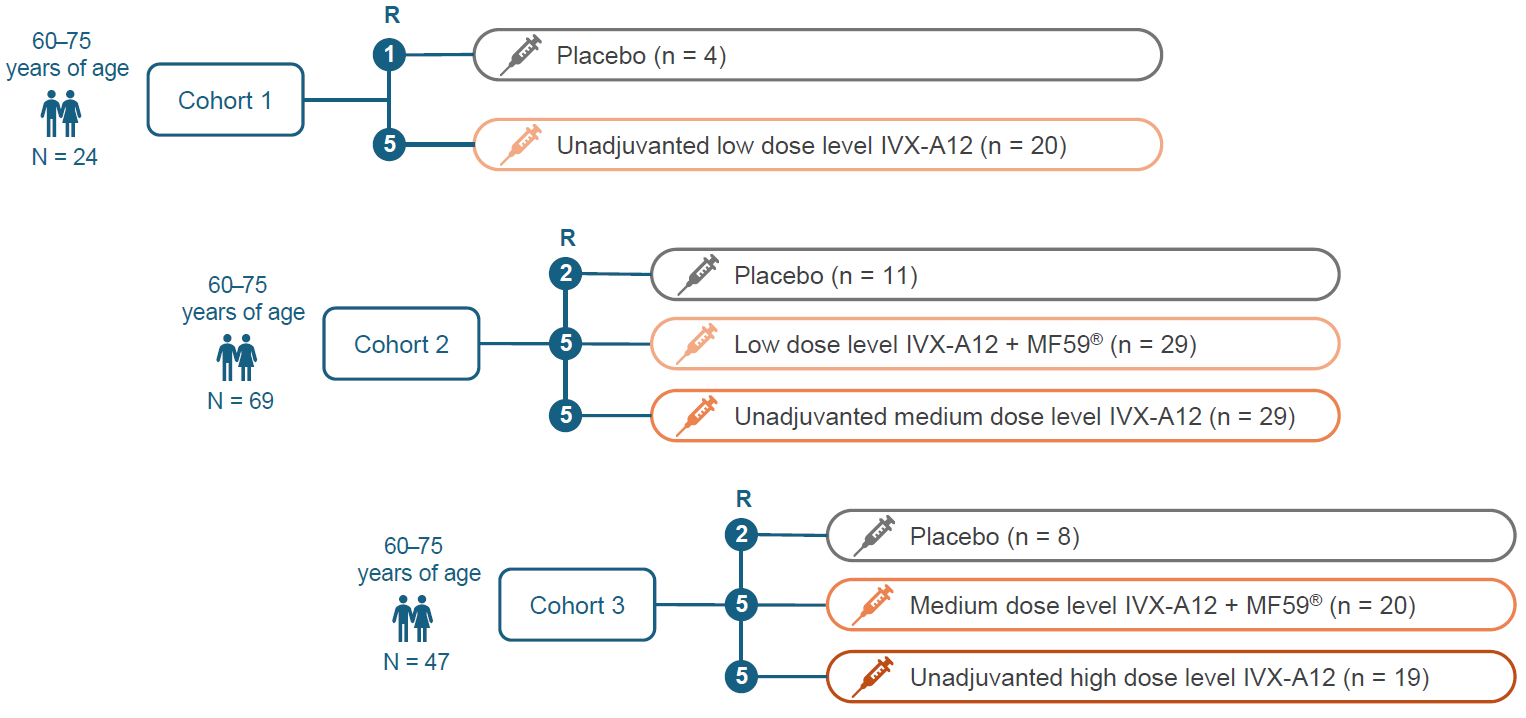


## Figure S2. RSV- and hMPV-specific nAb titers in participants with baseline nAb titers in the first tertile up to Day 365

nAb titers were assessed prior to vaccination on Day 0 (baseline), and post-vaccination on Days 7, 28, 180 and, 365 in the per-protocol set (N = 122); data for n = 7 participants were excluded at specific timepoints, as summarized in the appendix (p9). GMFRs at each timepoint were calculated from baseline. IVX-A12 dose levels were defined as follows: low = 75 µg RSV/75 µg hMPV; medium = 75 µg RSV/150 µg hMPV; high = 75 µg RSV/225 µg hMPV. See **Table S2** for a full listing of GMTs and GMFRs by baseline nAb titer tertile.
CI, confidence intervals; GMFR, geometric mean fold-rise; GMT, geometric mean titer; hMPV, human metapneumovirus; IU, international units; nAb, neutralizing antibody; RSV, respiratory syncytial virus; SRR, seroresponse rate.


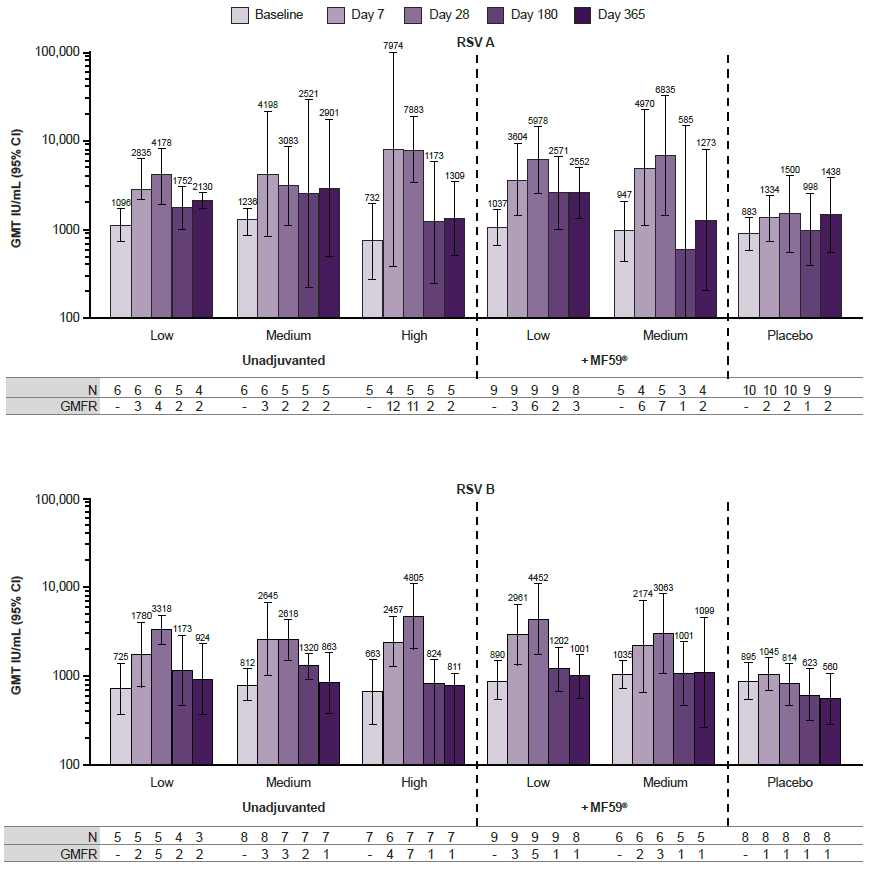


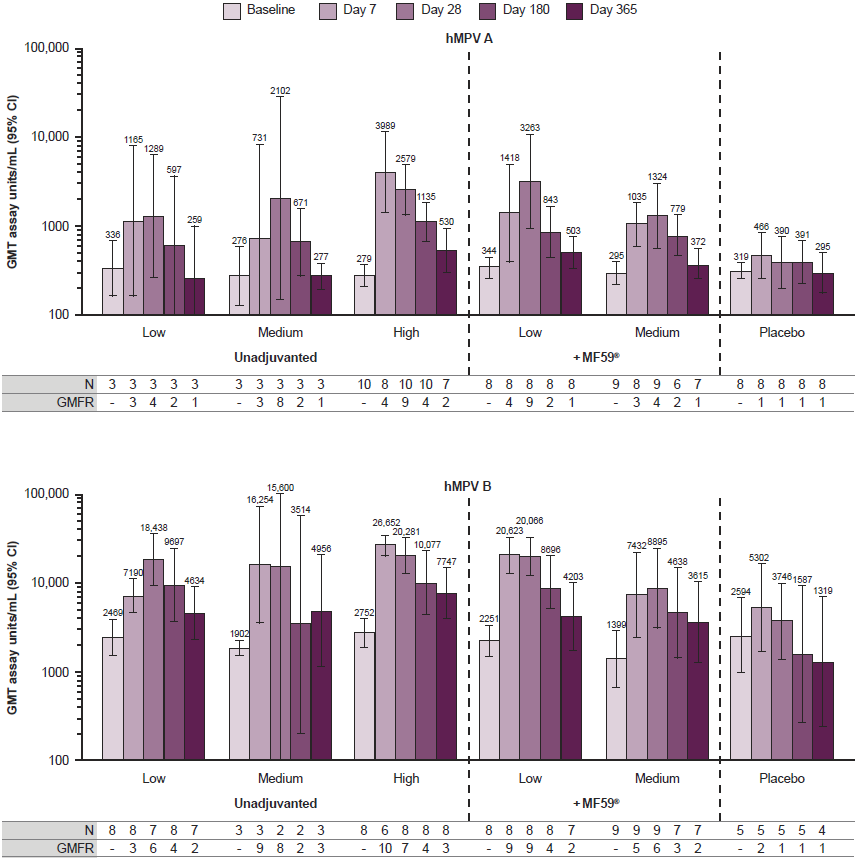


## Figure S3. VLP core-specific IgG titers up to Day 365

VLP core-specific IgG titers were assessed prior to vaccination on Day 0 (baseline), and post-vaccination on Days 28, 180, and 365 in the per-protocol set (N = 122); data for n = 7 participants were excluded at specific timepoints, as summarized in the appendix (p9). GMFRs at each timepoint were calculated from baseline. IVX-A12 dose levels were defined as follows: low = 75 µg RSV/75 µg hMPV; medium = 75 µg RSV/150 µg hMPV; high = 75 µg RSV/225 µg hMPV.


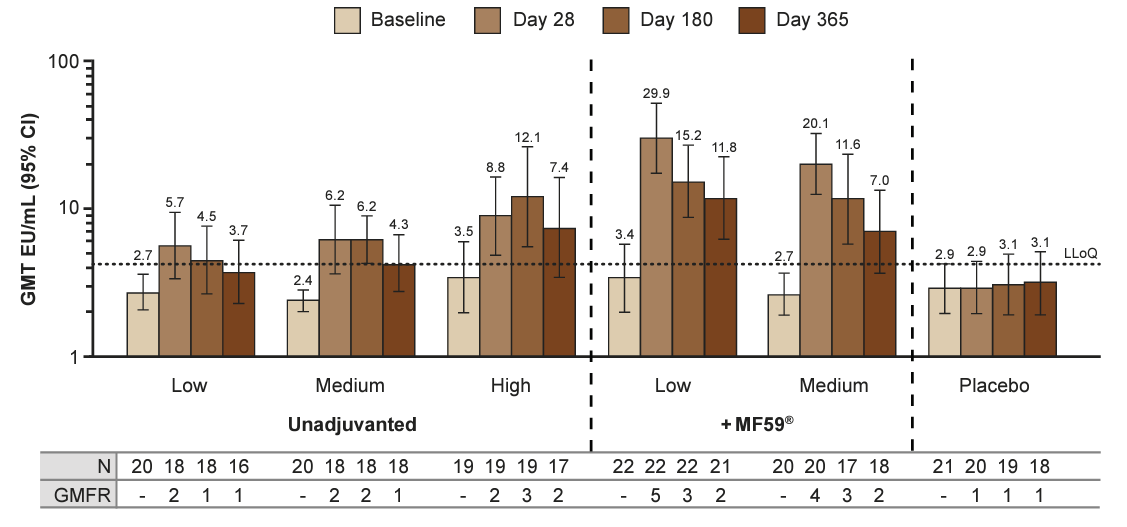
CI, confidence intervals; ELISA, enzyme-linked immunosorbent assay; EU, ELISA units; GMFR, geometric mean fold-rise; GMT, geometric mean titer; hMPV, human metapneumovirus; IgG, immunoglobulin G; LLoQ, lower limit of quantification; RSV, respiratory syncytial virus; VLP, virus-like particle.

# Supplementary References

1. US Food and Drug Administration (FDA). Guidance for Industry: Toxicity Grading Scale for Healthy Adult and Adolescent Volunteers Enrolled in Preventive Vaccine Clinical Trials. 2007. Available at: <https://www.fda.gov/media/73679/download>. Last accessed 25 September 2024.
